# *Hsf* and *Hsp* gene families in *Populus*: genome-wide identification, organization and correlated expression during development and in stress responses

**DOI:** 10.1186/s12864-015-1398-3

**Published:** 2015-03-14

**Authors:** Jin Zhang, Bobin Liu, Jianbo Li, Li Zhang, Yan Wang, Huanquan Zheng, Mengzhu Lu, Jun Chen

**Affiliations:** State Key Laboratory of Tree Genetics and Breeding, Key Laboratory of Tree Breeding and Cultivation of the State Forestry Administration, Research Institute of Forestry, Chinese Academy of Forestry, Beijing, 100091 China; Co-Innovation Center for Sustainable Forestry in Southern China, Nanjing Forestry University, Nanjing, 210037 China; College of Forestry, Fujian Agriculture and Forestry University, Fuzhou, Fujian 350002 China; State Key Laboratory of Crop Biology, Shandong Key Laboratory of Crop Biology, College of Life Sciences, Shandong Agricultural University, Tai’an, Shandong 271018 China; Department of Biology, McGill University, 1205 Dr Penfield Avenue, Montreal, Quebec H3A 1B1 Canada

**Keywords:** Coexpression, Expression analysis, Gene family, Heat shock factor (Hsf), Heat shock protein (Hsp), *Populus*

## Abstract

**Background:**

Heat shock proteins (Hsps) are molecular chaperones that are involved in many normal cellular processes and stress responses, and heat shock factors (Hsfs) are the transcriptional activators of *Hsps. Hsfs* and *Hsps* are widely coordinated in various biological processes. Although the roles of *Hsfs* and *Hsps* in stress responses have been well characterized in *Arabidopsis*, their roles in perennial woody species undergoing various environmental stresses remain unclear.

**Results:**

Here, a comprehensive identification and analysis of *Hsf* and *Hsp* families in poplars is presented. In *Populus trichocarpa*, we identified 42 paralogous pairs, 66.7% resulting from a whole genome duplication. The gene structure and motif composition are relatively conserved in each subfamily. Microarray and quantitative real-time RT-PCR analyses showed that most of the *Populus Hsf* and *Hsp* genes are differentially expressed upon exposure to various stresses. A coexpression network between *Populus Hsf* and *Hsp* genes was generated based on their expression. Coordinated relationships were validated by transient overexpression and subsequent qPCR analyses.

**Conclusions:**

The comprehensive analysis indicates that different sets of *PtHsps* are downstream of particular *PtHsfs* and provides a basis for functional studies aimed at revealing the roles of these families in poplar development and stress responses.

**Electronic supplementary material:**

The online version of this article (doi:10.1186/s12864-015-1398-3) contains supplementary material, which is available to authorized users.

## Background

During their growth, plants are subjected not only to abiotic stresses, such as irradiation, temperature, salinity, and drought, but also biotic stresses, such as herbivore and pathogen attacks. These stress factors can simultaneously act on the plants causing cell injury and producing secondary stresses [1,2]. As sessile organisms, plants cannot move to avoid these stresses, and thus have developed mechanisms, such as morphological adaptation, to tolerate these stresses [3].

Along with other stresses, heat stress can trigger the expression of certain genes that were not expressed under “normal” conditions [4-6]. Heat shock proteins (Hsps) accumulate when the expression of their genes is triggered by heat, as well as other stresses [7-9]. Hsps are molecular chaperones that regulate the folding, localization, accumulation, and degradation of protein molecules in both plant and animal species [10]. The expression of *Hsps* is controlled and regulated by specific types of transcription factors called heat shock factors (Hsfs), which normally exist as inactive proteins [11].

Plant Hsps are classified into five families based on their approximate molecular weights: small Hsp (sHsp), Hsp60, Hsp70, Hsp90, and Hsp100. Genes encoding Hsfs and Hsps have been well characterized in some model plants, such as *Arabidopsis* and rice [12,13]. In *Arabidopsis*, 21, 27, 18, 18, 7, and 4 genes have been identified as *Hsf*, *sHsp*, *Hsp60*, *Hsp70*, *Hsp90*, and *Hsp100* family members, respectively [13-19].

To date, various stress responses and functions of Hsf and Hsp members have been reported. In *Arabidopsis*, HsfA1a, HsfA1b, and HsfA1d act as the main positive regulators of heat shock response [20]. *Arabidopsis HsfA2* was significantly increased under high light and heat stress or by H_2_O_2_ treatment. It is the key regulator in the induction of the defense system under several types of environmental stresses [21,22]. The cytosolic class I *sHsp* in *Rosa chinensis*, *RcHsp17.8*, was induced by heat, cold, salt, drought, osmotic, and oxidative stresses [23]. A plastid nucleoid-localized sHsp (Hsp21) interacts with the plastid nucleoid protein pTAC5 and is essential for chloroplast development in *Arabidopsis* under heat stress [24]. Hsp60 proteins as chaperones participate in the folding and aggregation of many proteins that are transported to organelles, such as chloroplasts and mitochondria [25,26]. *Arabidopsis hsp60* mutants showed defects in chloroplast, embryo, and seedling development and also increased cell death [27,28]. Unlike other family members that are mainly expressed when the organism is subjected to environmental assaults, Hsp70s play essential functions in facilitating refolding and proteolytic degradation of abnormal proteins under both normal and stress conditions [29,30]. Hsp100 proteins belong to the Caseinolytic protease (Clp) family, which forms an ATP-dependent protease complex that is able to hydrolyze casein [19,31-33]. Tomatoes that have an antisense suppression of *Hsp100/ClpB* are more heat sensitive [34]. Moreover, Lee *et al.* [19] noted that *Arabidopsis* mutant plants containing a chloroplast-localized *Hsp100/ClpB-p* knockout turned yellow after heat treatment. Hsps were not only transcriptionally regulated by Hsfs, but could also regulate the activity of Hsfs through a feedback loop caused by their physical interaction. In tomato, Hsp70 and Hsp90 regulate the Hsfs’ function by directly interacting with Hsfs. Hsp70 represses the activity of HsfA1 and HsfB1, while the DNA binding activity of HsfB1 is stimulated by Hsp90 in tomato [35]. Above all, Hsfs and Hsps play crucial roles in plant development and various stress tolerances.

As perennial species, poplars undergo seasonal variations and various environmental stresses frequently. The completion of the *Populus trichocarpa* genome sequencing project in 2006 makes it an ideal genetic model for studying tree development and physiology [36]. To date, 28 *Populus Hsfs* have been reported based on the V2.2 *P. trichocarpa* genome database [11]. In our previous study, we have reported the subcellular localization and expression, under various abiotic stresses, of 10 *Hsp90* genes identified from the V3.0 *P. trichocarpa* genome database [37]. However, the other Hsp families in poplar remain unclear, and little is known about the transcriptional regulatory relationships between *Populus* Hsfs and Hsps. Here, we provide a comprehensive analysis of the gene organization and expression of *Populus Hsfs* and other *Hsp* genes, including *sHsp*, *Hsp60*, *Hsp70* and *Hsp100*, under different abiotic stresses. A complex transcriptional regulatory network between *Populus Hsfs* and *Hsps* has been generated based on their transcription patterns in poplar.

## Results

### Identification and phylogenetic analysis of the *Hsf* and *Hsp* gene families in poplar

After automated database searching and a manual review, 118 genes were identified as members of the *Hsf* and *Hsp* families, including *sHsp*, *Hsp60*, *Hsp70*, and *Hsp100*, of *P. trichocarpa*. The *Hsf* and *Hsp* gene families, *PtHsf* and *PtHsp*, respectively, in poplar were relatively large compared with those in *Arabidopsis* and rice. The numbers of identified genes in the *Hsf*, *sHsp*, *Hsp60*, *Hsp70*, and *Hsp100* families of *P. trichocarpa* were 28, 37, 28, 20, and 5, respectively (Table 1). The subcellular localization predictions suggested that the PtHsfs are targeted to the nucleus, while PtHsps are localized to various cytosolic organelles. Detailed information on the *Hsf* and *Hsp* genes in *P. trichocarpa*, including their gene IDs and the characteristics of their encoded proteins, are listed in Additional file 1: Table S1 and Additional file 2: Table S2.

**Table 1:** **Numbers of** ***Hsf*** **and** ***Hsp*** **genes in** ***Arabidopsis*** **,** ***Populus*** **and rice**

	***A. thaliana***	***O. sativa***	***P. trichocarpa***
*Hsf*	21	25	28
*sHsp*	27	29	37
*Hsp60*	18	20	28
*Hsp70*	18	26	20
*Hsp90*	7	9	10
*Hsp100*	4	5	5

**Figure 1:**
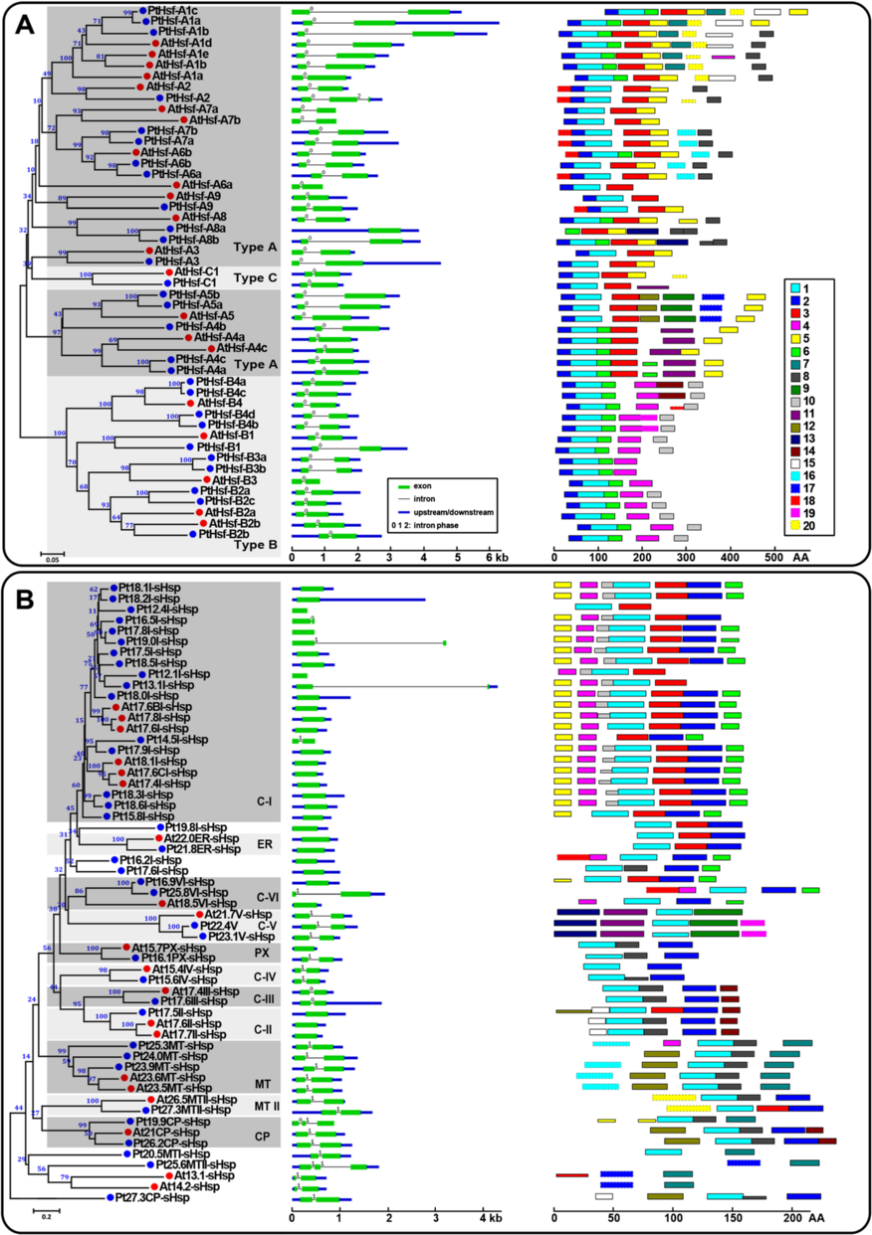
**Phylogenetic relationships, gene structures and motif compositions of** ***Hsf*** **and** ***sHsp*** **family members in** ***A. thaliana*** **(At) and** ***P. trichocarpa*** **(Pt).** Multiple alignment of Hsf **(A)** and sHsp **(B)** proteins from *A. thaliana* (At) and *P. trichocarpa* (Pt) was performed using MEGA 5.0 by the Neighbor-Joining (NJ) method with 1000 bootstrap replicates (left panel). Exon/intron structures of the *Hsf* and *sHsp* genes are shown in the middle panel. Green boxes represent exons and black lines represent introns. The numbers indicate the splicing phases of the *Hsf* and *sHsp* genes: 0, phase 0; 1, phase 1; and 2, phase 2. A schematic representation of conserved motifs (obtained using MEME) in Hsf and sHsp proteins is displayed in the right panel. Different motifs are represented by different colored boxes. Details of the individual motifs are in the Additional file 4: Table S4 and Additional file 5: Table S5.

**Figure 2:**
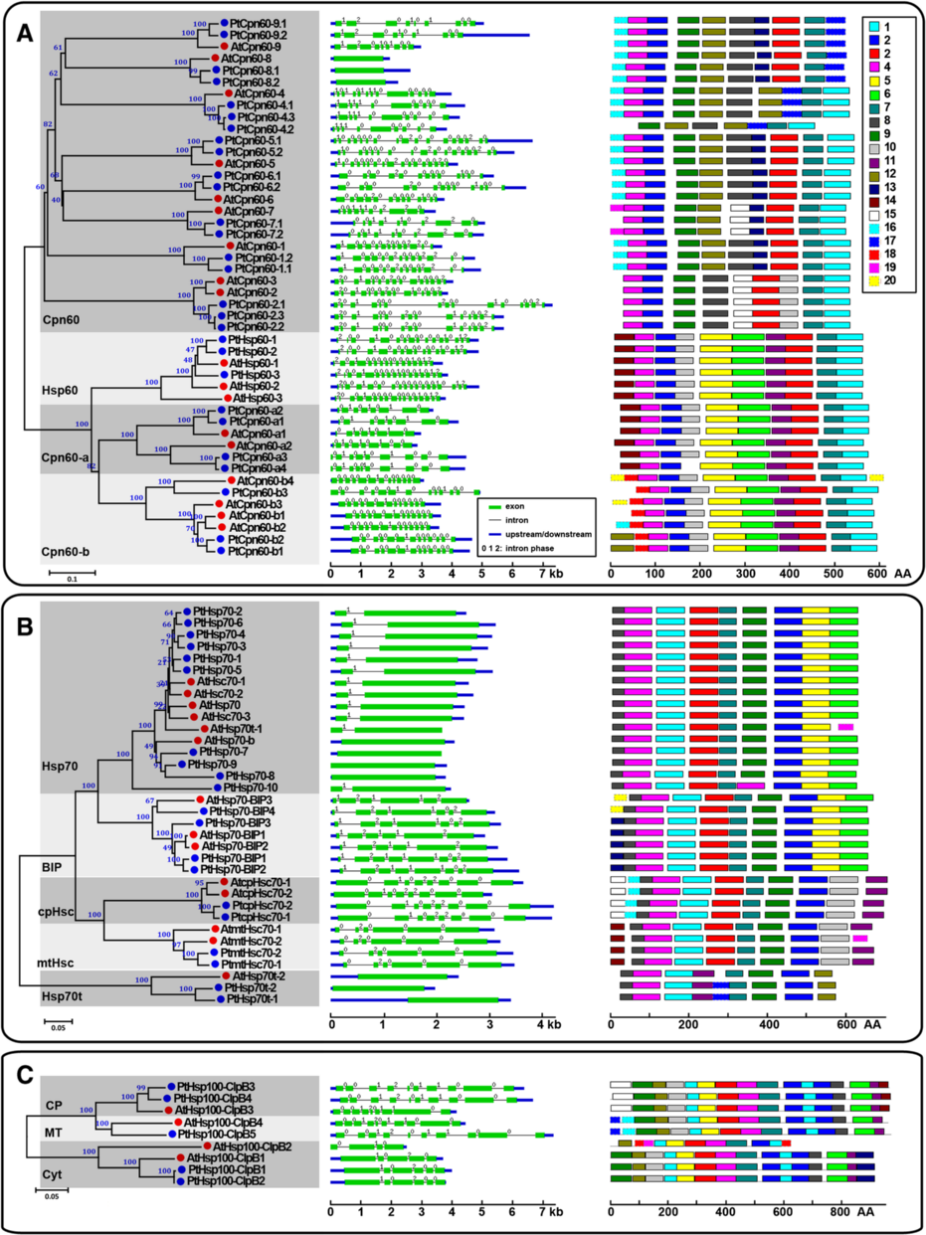
**Phylogenetic relationships, gene structures and motif compositions of** ***Hsp60*** **,** ***Hsp70*** **and** ***Hsp100*** **family members in** ***A. thaliana*** **(At) and** ***P. trichocarpa*** **(Pt).** Multiple alignment of Hsp60 **(A)**, Hsp70 **(B)** and Hsp100 **(C)** proteins from *A. thaliana* (At) and *P. trichocarpa* (Pt) was performed using MEGA 5.0 by the Neighbor-Joining (NJ) method with 1000 bootstrap replicates (left panel). Exon/intron structures of the *Hsp60* **(A)**, *Hsp70* **(B)** and *Hsp100* **(C)** genes are shown in the middle panel. Green boxes represent exons and black lines represent introns. The numbers indicate the splicing phases of the *Hsp60* **(A)**, *Hsp70* **(B)** and *Hsp100* **(C)** genes: 0, phase 0; 1, phase 1; and 2, phase 2. A schematic representation of conserved motifs (obtained using MEME) in Hsp60 **(A)**, Hsp70 **(B)** and Hsp100 **(C)** proteins is displayed in the right panel. Different motifs are represented by different colored boxes. Details of the individual motifs are in the Additional file 6: Table S6, Additional file 7: Table S7 and Additional file 8: Table S8.

To evaluate the evolutionary relationship of the Hsf and Hsp proteins, a phylogenetic analysis of each family was performed based on the full-length amino acid sequences from both *P. trichocarpa* and *Arabidopsis* (Figures 1 and 2, left panel). Each family could be classified into different subfamilies. The *PtHsf* family contains three subfamilies: type A (17 genes), type B (10 genes), and type C (1 gene). However the subfamilies in each of the *PtHsp* families could be assigned based on the proteins’ predicted subcellular localization. The *sHsp* family was classified into cytosolic, endoplasmic reticulum (ER), peroxisome (PX), chloroplast (CP), and mitochondrial (MT) subfamilies in *P. trichocarpa*. There are six groups of cytosolic *sHsp* genes, C-I, C-II, C-III, C-IV, C-V, and C-VI, and two groups of mitochondrial *sHsp* genes, MT and MT II, in *P. trichocarpa*. Notably, the C-I *sHsp* group in the genome of *P. trichocarpa* is large, containing 19 genes compared with 6 in *Arabidopsis* (Figure 1). The *Hsp60* family was divided into four subfamilies in *P. trichocarpa*: cytosol-localized *Cpn60* (18 genes), mitochondrion-localized *Hsp60* (3 genes), and chloroplast-localized *Cpn60-a* (4 genes) and *Cpn60-b* (3 genes). The *Hsp70* family contains genes encoding 10 cytosolic Hsp70s, 4 binding proteins (*BIPs*, *Hsp70* homologs in the ER), 2 plastid Hsp70s (cpHsc70s), 2 mitochondrial Hsp70s (mtHsc70s), and 2 truncated Hsp70s (Hsp70ts). The *Hsp100* family can be divided into three classes in *P. trichocarpa*, cytoplasmic (Cyt, 2 genes), chloroplastic (CP, 2 genes), and mitochondrial (MT, 1 gene) (Figure 2).

### Structure of *Hsf* and *Hsp* genes and conserved motifs of Hsf and Hsp proteins in poplar

To gain further insights into the structural diversity of *Hsf* and *Hsp* genes in *P. trichocarpa*, we compared the exon/intron organization in the coding sequences between individual *Hsf* and *Hsp* genes of *P. trichocarpa* and *Arabidopsis* (Figures 1 and 2, middle panel). Most closely related members in the same *Hsf* or *Hsp* subfamilies shared similar intron numbers or exon length. In the *PtHsp70* family, cytosolic *Hsp70s* have zero or one intron, ER-localized *BIPs* have six introns, mitochondrion-localized *mtHsc70s* have five introns and chloroplast-localized *cpHsc70s* have seven introns, while truncated *Hsp70ts* have no introns (Figure 2B). Interestingly, two cytosol-localized *Hsp60* members, *PtCpn60-8.1* and *PtCpn60-8.2*, have no introns in their coding regions while the other *P. trichocarpa Hsp60s* contain several introns (8–16) (Figure 2A). We then compared the intron phases with respect to the codons. The intron phases were remarkably well conserved within the same subfamilies in the detected *PtHsf* and *PtHsp* families.

We further detected the exon/intron structure of 42 paralogous pairs of the *PtHsf* and *PtHsp* genes (10, 9, 12, 9, and 2 pairs in *P. trichocarpa Hsf*, *sHsp*, *Hsp60*, *Hsp70*, and *Hsp100* gene families, respectively, Additional file 3: Table S3). Although 36 paralogous pairs showed conserved intron numbers and gene lengths, one *Hsf* gene pair (*PtHsfA8a/PtHsfA8b*), four *sHsp* pairs (*Pt17.8I-sHsp/Pt19.0I-sHsp*, *Pt12.2I-sHsp/Pt13.1I-sHsp*, *Pt14.5I-sHsp/Pt17.9I-sHsp*, and *Pt16.9VI-sHsp/Pt25.8VI-sHspI*), and one *Hsp60* pair (*PtCpn60-a3/PtCpn60-a4*) exhibited certain degrees of variation (Figures 1 and 2).

We then used the Multiple Expectation Maximization for Motif Elicitation (MEME) [38] to predict the conserved motifs shared among the related proteins within these families. In each family, 20 putative motifs were identified. The details of these motifs are listed in Additional file 4: Tables S4; Additional file 5: Tables S5; Additional file 6: Tables S6; Additional file 7: Tables S7 and Additional file 8: Tables S8. Most of the closely related members in the phylogenetic tree shared common motif compositions with each other (Figures 1 and 2, right panel).

### Chromosomal location and duplication of *Hsf* and *Hsp* genes in poplar

**Figure 3:**
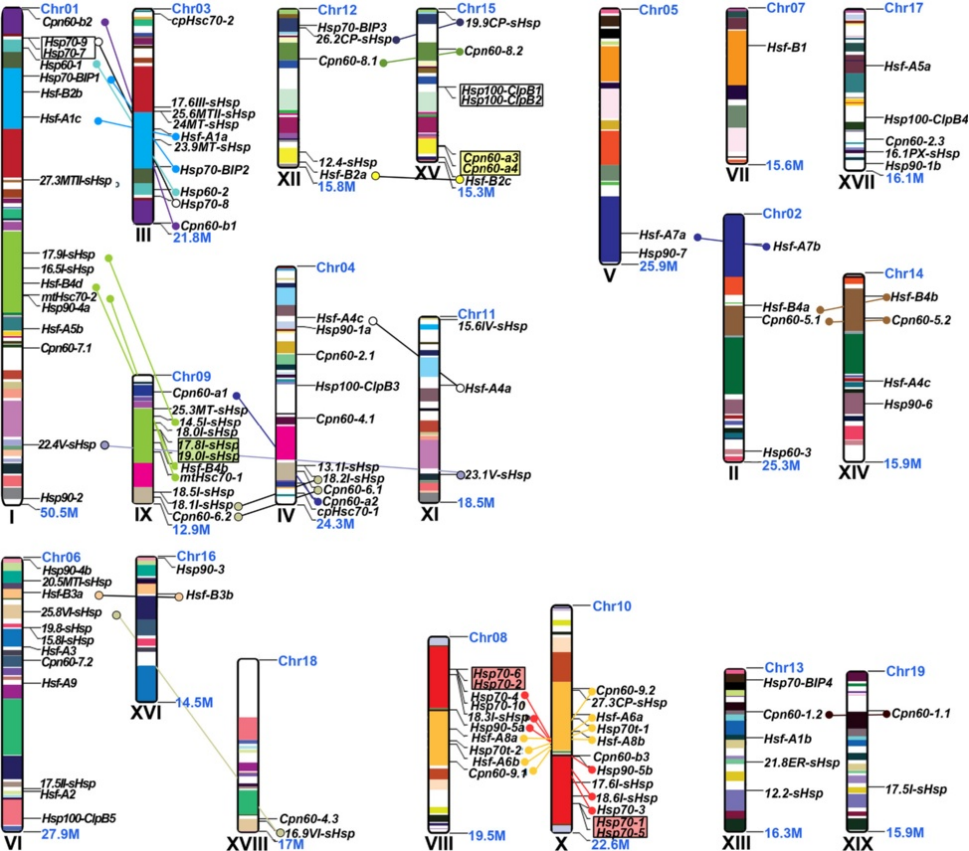
**Chromosomal locations of** ***Populus Hsfs*** **and** ***Hsps*** **.** 114 of 118 *Hsf* and *Hsp* genes are mapped to 19 chromosomes. The schematic diagram of *Populus* genome-wide chromosome organization arisen from the whole genome duplication event was adapted from Tuskan *et al.* [36] and Chai *et al.* [54]. Homologous blocks derived from segmental duplication are indicated using the same colors. Small circles connected by colored line indicate corresponding sister gene pairs.

To investigate the expansion of *Hsf* and *Hsp* genes in *P. trichocarpa*, the identified *PtHsf* and *PtHsp* genes were plotted on the chromosomes. Of 118 *PtHsf* and *PtHsp* genes, 114 were distributed on 19 chromosomes, while only four genes (*PtHsf-C1*, *Pt16.2I-sHsp*, *PtCpn60-2.2*, and *PtCpn60-4.2*) localized to unassembled genomic sequence scaffolds (Figure 3). The distributions of *Hsf* and *Hsp* genes among the chromosomes appeared to be uneven in *P. trichocarpa*: chromosome (chr) V, VII, XVI, XVIII, and XIX contain only one or two *Hsf* and *Hsp* genes, while relatively high densities of *Hsf* and *Hsp* genes were discovered on chr I, III, VI, VIII, IX, and X. In particular, *Hsfs* and *Hsps* were clustered on the duplicated fragments of chr VIII and X in *P. trichocarpa*.

The genome of *P. trichocarpa* has experienced at least two whole genome duplication (WGD) events, followed by a series of chromosomal reorganizations involving reciprocal tandem/terminal fusions and translocations [36]. Approximately 84.7% (100 of 118) *Hsf* and *Hsp* genes were located in the replicated region, while 44 genes lacked copies on the corresponding duplicated blocks. A chromosome region containing two or more genes within 200 kb can be defined as a gene cluster [39]. The gene clusters were distributed unevenly among *Hsf* and *Hsp* gene families. The *sHsp*, *Hsp60*, *Hsp70*, and *Hsp100* families contain five, one, three, and one clusters, respectively (Figure 3 and Additional file 3: Table S3). The smallest tandem duplication clusters consisted of only two genes and the largest cluster, in the *Hsp70* family, had five genes. Interestingly, none of the *Hsf* genes were represented in tandem clusters.

Among the 42 paralogous pairs of the *Populus Hsf* and *Hsp* families, 66.7% gene pairs (28 of 42 pairs) were generated by whole genome duplication and 19% (8 of 42 pairs) by tandem duplication (Additional file 3: Table S3). To verify whether Darwinian positive selection was involved in the *Hsf* and *Hsp* gene divergence after duplication, the nonsynonymous (*K*a) versus synonymous (*K*s) substitution rate ratios were calculated for the 42 paralogous pairs [40]. A *K*a/*K*s ratio significantly lower than 0.5 suggests a purifying selection for both duplicates [41]. The summary of *K*a/*K*s for the 42 *Hsf* and *Hsp* gene paralogous pairs is shown in Additional file 3: Table S3.

### Expression patterns of *Populus Hsf* and *Hsp* genes

Publicly available Expressed Sequence Tags (ESTs) provide a useful tool to survey gene expression profiles using a digital northern blot [42]. We conducted a preliminary expression analysis of *Hsf* and *Hsp* genes by counting the frequencies of ESTs obtained from different tissues and under various growth conditions in different *Populus* cDNA libraries (Additional file 9: Figure S1). A complete search of the digital expression profiles from PopGenIE (http://popgenie.org/) [43] yielded 77 *Populus Hsf* and *Hsp* genes in the cDNA libraries. The frequencies of these ESTs were relatively low, and most *Hsf* and *Hsp* genes were represented by only one EST in the cDNA libraries. Nevertheless, these expression profiles suggested that most of the *PtHsf* and *PtHsp* genes had broad expression patterns across different tissues.

**Figure 4:**
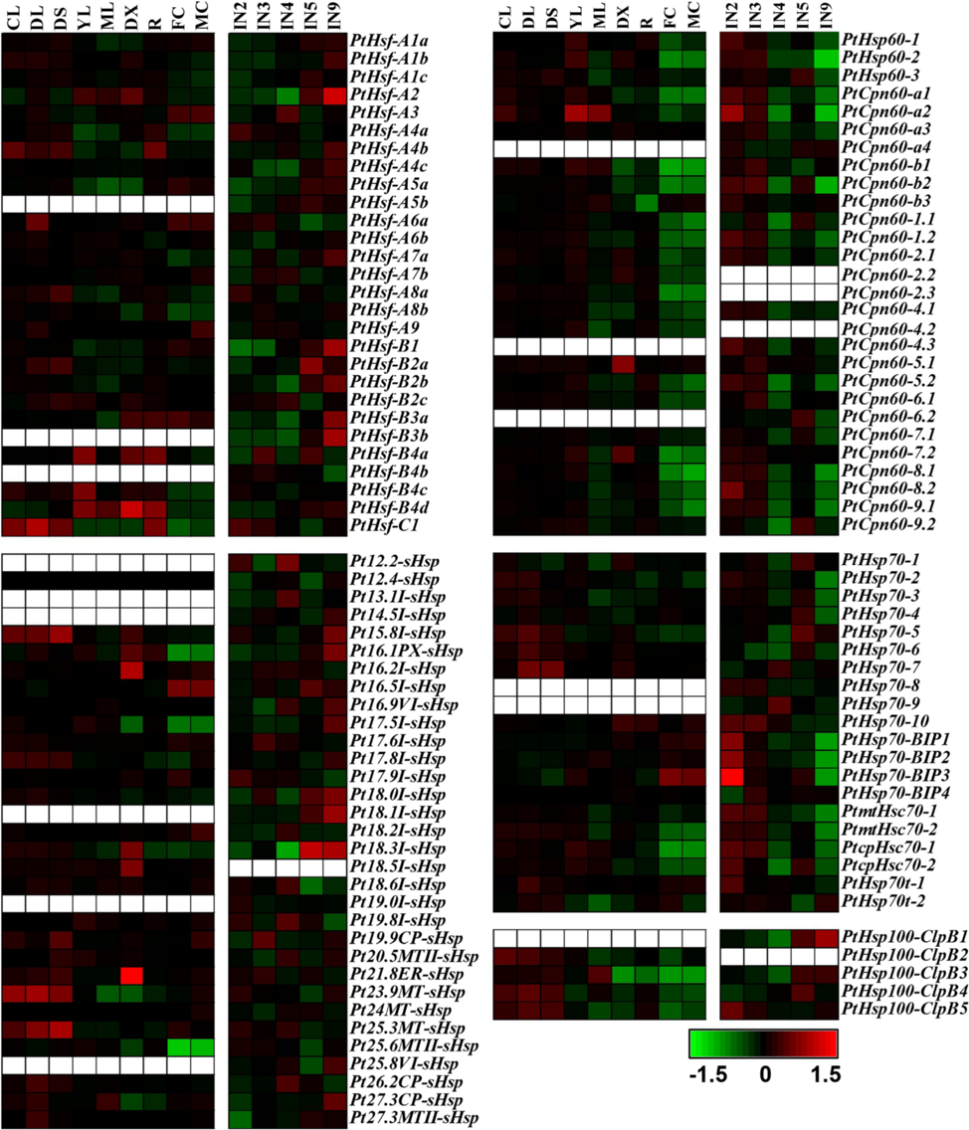
**Expression profiles of** ***Populus Hsfs*** **and** ***Hsps*** **across different tissues.** Heatmap showing expression of *Hsf* and *Hsp* genes across various tissues and different stem development/growth stages. The Affymetrix microarray data (GSE13990) and the NimbleGen microarray data (GSE13043) were obtained from NCBI Gene Expression Omnibus (GEO) database. CL, continuous light-grown seedling; DL, etiolated dark-grown seedling transferred to light for 3 h; DS, dark-grown seedlings; YL, young leaf; ML, mature leaf; R, root; DX, differentiating xylem; FC, female catkins; MC, male catkins; IN2-IN5, and IN9, stem internode 2 to internode 5, and internode 9. Background corrected expression intensities were log transformed and visualized as heatmaps (see Methods). Color scale represents log2 expression values, green represents low level and red indicates high level of transcript abundance. Blank represents a gene has no corresponding probe sets in the microarray data.

We then investigated the global expression profiles of *Hsf* and *Hsp* genes by examining previously published microarray data in poplars. At first, Affymetrix (GSE13990) [44] and a Nimblegen (GSE13043) [45] microarray data from Gene Expression Omnibus [46] were used to analyze the expression patterns of *Hsf* and *Hsp* genes in different tissues. Most *Populus Hsf* and *Hsp* genes were detected in the two different platforms. The majority of *Hsf* and *Hsp* genes showed a tissue-specific expression pattern (Figure 4). Four *sHsps* (*Pt16.2I-sHsp*, *Pt18.3I-sHsp*, *Pt18.5I-sHsp*, and *Pt21.8ER-sHsp*) and two *Hsp60s* (*PtCpn60-5.1* and *PtCpn60-7.2*) had high transcript levels in the differentiating xylem. Two *Hsfs* (*PtHsf-A3* and *PtHsf-B3a*), one *sHsp* (*Pt16.5I-sHsp*) and one *Hsp70* (*PtHsp70-BIP3*) were preferentially expressed in male and female catkins, but almost all of the *Hsp60s* had low transcript levels in catkins (Figure 4). During stem development, 9 *Hsfs,* 13 *sHsps*, and 2 *Hsp100s* had high levels in the basal stem undergoing the secondary growth (internode 9). In comparison, most *Hsp60* and *Hsp70* genes showed high expression levels in the upper stem (internode 2 and 3) (Figure 4).

**Figure 5:**
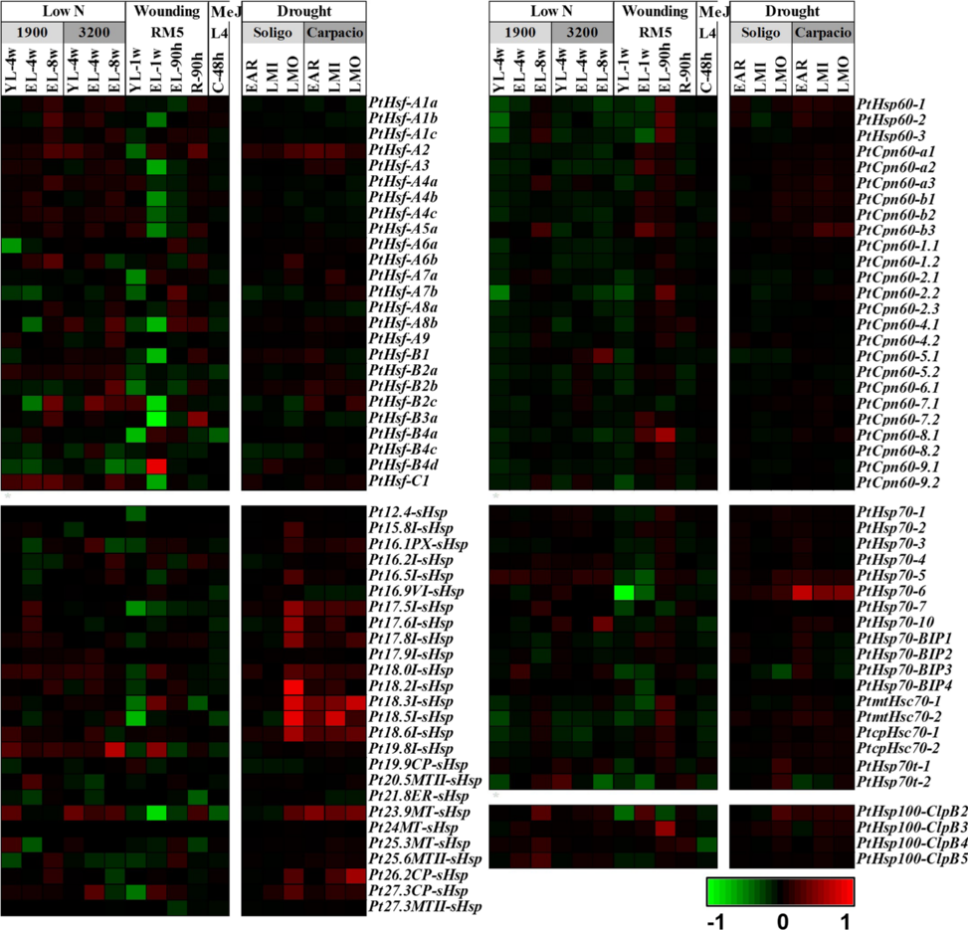
**Differential expression of** ***Populus Hsfs*** **and** ***Hsps*** **under different abiotic stresses.** Heatmap showing expression of *Hsf* and *Hsp* genes across various tissues and genotypes analyzed. Microarray data under the series accession number GSE16786 (for low N, wounding, and MeJ treatment) and GSE17230 (for drought treatment) was obtained from NCBI GEO database. Genotypes analyzed included: *P. fremontii* × *P. angustifolia* clones 1979, 3200, and RM5, *P. tremuloides* clones L4, and *P. deltoids* clones Soligo and Carpaccio. Tissues analyzed included: YL, young leaves; EL, expanding leaves; R, root tips; C, suspension cell cultures. Stress treatments included: low N, nitrogen limitation; wounding, sampled either one week or 90 hours after wounding; MeJ, Methyl Jasmonate elicitation; EAR, early response (EAR) to water deficit by 36 hours; LMI, long-term (10-day) response to mild stress with soil relative extractable water (REW) at 20–35%; LMO, long-term (10-day) response to moderate stress with soil REW at 10–20%. Background corrected expression intensities were log transformed and visualized as heatmaps (see Methods). Color scale represents log2 expression values, green represents low level and red indicates high level of transcript abundances.

To explore the possible roles of *Populus Hsf* and *Hsp* genes in response to various abiotic stresses, we then analyzed their expression patterns under heat, drought, low nitrogen level, mechanical wounding, and methyl jasmonate (MeJ) treatment. Four *Hsf* genes (*PtHsf-A2*, *PtHsf-A6b PtHsf-B2c*, and *PtHsp-C1*), three *sHsps* (*Pt18.0I-sHsp*, *Pt19.8I-sHsp*, and *Pt23.9MT-sHsp*), one *Hsp70* (*PtHsp70-5*), and one *Hsp100* (*PtHsp100-ClpB2*) were up-regulated under nitrogen deprivation in both genotypes 1979 and 3200. Mechanical wounding caused the up-regulation of four *Populus Hsfs* in expanding leaves at 90 h after wounding, followed by a down-regulation at 1 week in young leaves and expanding leaves. In cell culture, the addition of MeJ led to the down-regulation of most *sHsp* and *Hsp70* genes (Figure 5). Notably, C-I *sHsp* genes were significantly up-regulated under drought stress in the two genotypes, while the other *Hsf* and *Hsp* genes were not significantly changed (Figure 5).

**Figure 6:**
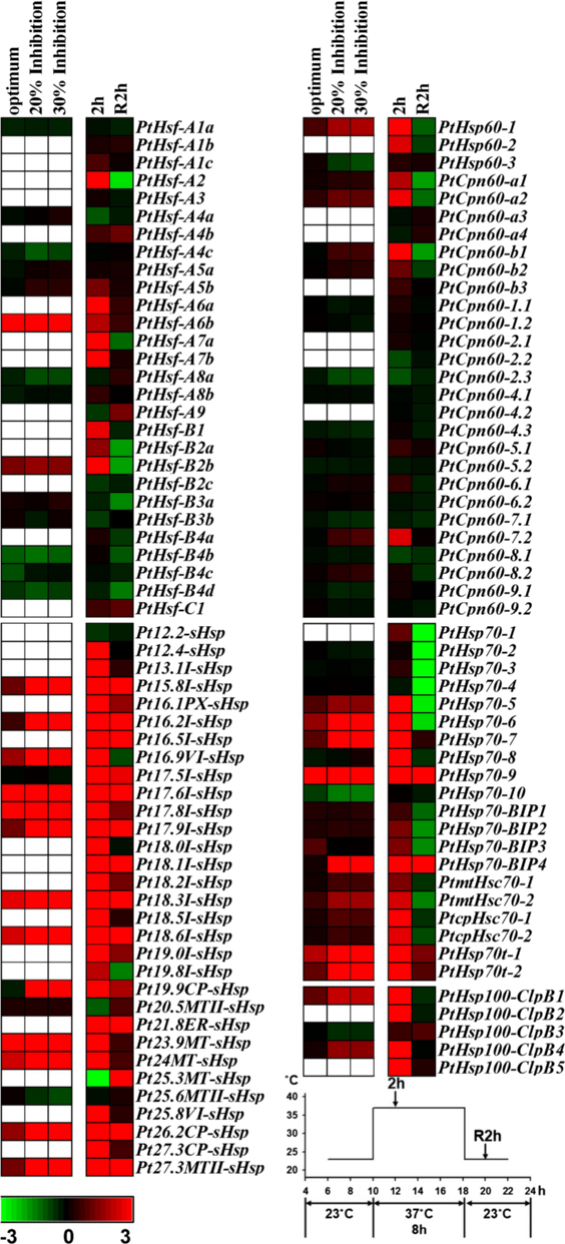
**Expression profiles of** ***Populus Hsfs*** **and** ***Hsps*** **under heat stress.** Heatmap showing expression of *Hsf* and *Hsp* genes under heat stress. Microarray data (GSE26199) obtained from NCBI GEO database and our RNA-seq data (unpublished, see Methods) were used to be analyzed. In GSE26199, the expression changes of *Hsf* and *Hsp* genes under photosynthetic optimum (31.75**°**C, temperature producing the maximum net CO_2_ assimilation rate), 20% inhibition of optimum (38.4**°**C) and 30% inhibition of optimum (40.5**°**C) relative to baseline (22**°**C, the growth temperature) were analyzed in *P. trichocarpa* leaves. In our RNA-seq data, the expression changes of *Hsf* and *Hsp* genes during heat stress (2 h, 2 hours after heat treatment at 37**°**C; R2h, 2 hours after recovery from 37**°**C) were analyzed in the 5th internode of *P. alba* × *P. glandulosa*. The expression level of genes was determined based on the value of FPKM (Fragments Per Kilobase of exon per Million fragments mapped). Details of the FPKM are shown in Additional file 10: Table S9. Color scale represents log2 expression values, green represents low level and red indicates high level of transcript abundance. Blank represents the gene has no corresponding probe sets in the microarray data.

In a previous study, the physiological conditions of poplars at temperatures between 22°C and 42°C were divided into four states based on photosynthetic activity: the baseline (22°C, the growth temperature), optimum (31.75°C, temperature producing the maximum net CO_2_ assimilation rate), 20% inhibition of optimum (38.4°C), and 30% inhibition of optimum (40.5°C) [47]. Most *sHsp* and *Hsp70* genes were induced when the temperature was increased to the optimum, and then continued to be induced when the photosynthesis was inhibited under heat stress, as shown using dataset GSE26199 in Figure 6 [47]. Because of the restrictions of the microarray probe sets, many *Hsf* and *Hsp* genes were not detected in GSE26199. To gather more information on the *Populus Hsf* and *Hsp* genes’ expression profiles under high temperatures, RNA-seq data from a hybrid poplar (*P. alba* × *P. glandulosa*) stem (internode 5) under the heat treatments (our unpublished data, Additional file 10: Table S9) were used to analyze the expression of *Populus Hsf* and *Hsp* genes. In the hybrid poplar, nine *Hsfs*, seven *Hsp60s*, and almost all of the *sHsps*, *Hsp70s*, and *Hsp100s* were induced 2 h after treated at 37°C (Figure 6). After a 2-h recovery period following the 37°C treatment, the expression levels of the induced *Hsfs*, *Hsp60s*, *Hsp70s*, and *Hsp100s* decreased, but not those of the *sHsps* (Figure 6).

### Confirmation of *Populus Hsf* and *Hsp* gene expression levels by qRT-PCR

**Figure 7:**
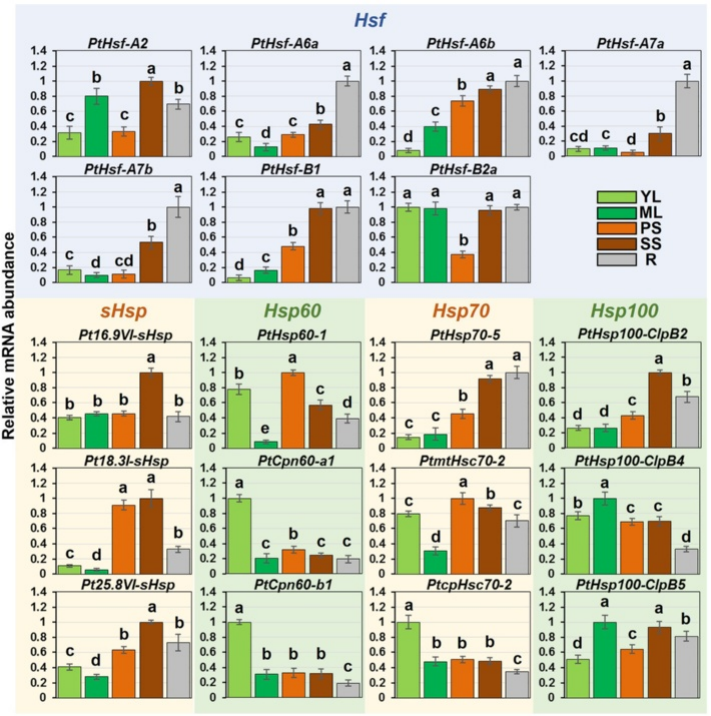
**qRT-PCR analysis of selected** ***Populus Hsfs*** **and** ***Hsps*** **in different tissues.** The relative mRNA abundance of selected seven *Hsfs*, three *sHsps*, three *Hsp60s*, three *Hsp70s* and three *Hsp100s* was normalized with respect to two reference genes *PtActin* and *PtTubulin* in five different tissues. Bars represent standard deviations (SD) of three technical replicates. YL, young leaves; ML, mature leaves; PS, primary stem; SS, secondary stem; R, roots. Bars with the same letter are not significantly different according to Duncan test and Fisher’s protected LSD test (*P* < 0.05).

To confirm the expression profiles of *Populus Hsf* and *Hsp* genes obtained from the microarray and RNA-seq data, a qRT-PCR analysis of selected *Hsf* and *Hsp* genes (seven *Hsf*, three *sHsp*, three *Hsp60*, three *Hsp70*, and three *Hsp100*) was performed on five different tissues (YL - young leaves, ML - mature leaves, PS - primary stem, SS - secondary stem, and R - root) of hybrid poplar. The gene expression patterns were mostly consistent with the microarray data (Figures 5 and 7).

**Figure 8:**
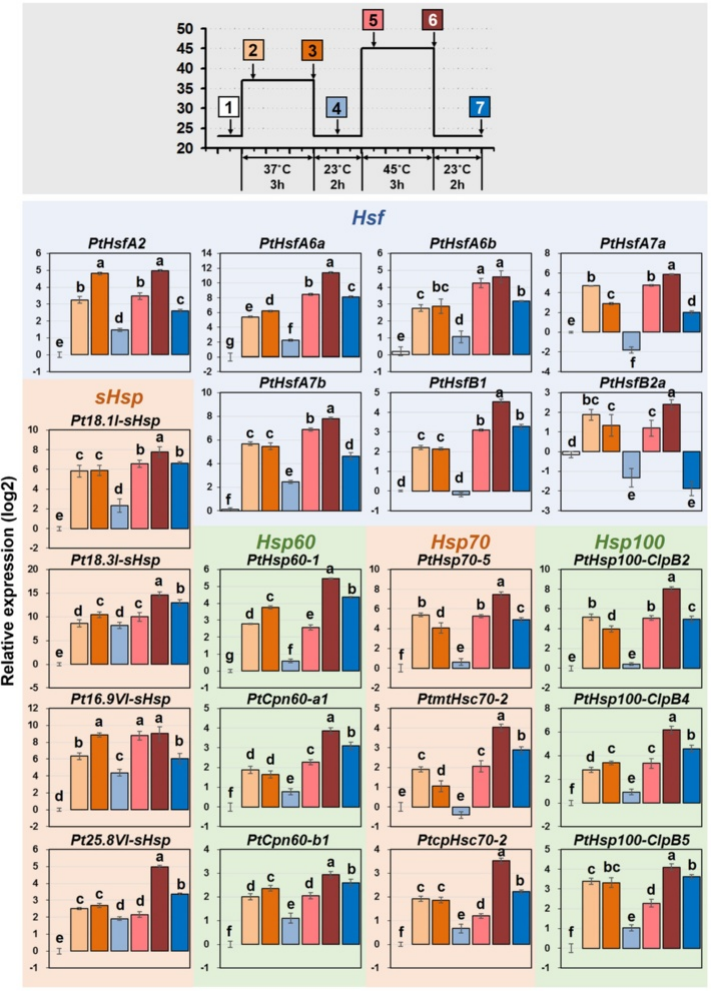
**qRT-PCR analysis of selected** ***Populus Hsfs*** **and** ***Hsps*** **under heat stress.** The relative mRNA abundance of selected seven *Hsfs*, four *sHsps*, three *Hsp60s*, three *Hsp70s*, and three *Hsp100s* during heat stress were quantified. Seedlings were heated to 37°C for 3 h (pretreatment), return to 23°C for 2 h, heated to 45°C for 3 h (treatment), and then allowed to 23°C for 2 h. 1, control; 2, 30 min after pretreatment at 37°C; 3, 2 h after pretreatment at 37°C; 4, 1 h after recovery at 23°C; 5, 30 min after treatment at 45°C; 6, 2 h after treatment at 45°C; 7, 2 h after recovery at 23°C. Relative expression represents log2 expression values. Bars with the same letter are not significantly different according to Duncan’s test and Fisher’s protected LSD test (*P* < 0.05).

We also analyzed the response of the selected *Hsf* and *Hsp* genes to high temperatures by qRT-PCR. A heat treatment composed a 37°C pretreatment for 3 h, a subsequent 45°C treatment for 3 h, and a 2-h recovery interval was performed (Figure 8) [37]. All of the selected *Hsf* and *Hsp* genes were induced immediately after 30 min in the 37°C pretreatment and 45°C treatment. The expression levels of the detected genes after the 45°C treatment were relatively high compared with that of the 37°C pretreatment. After a 2-h recovery from the 37°C pretreatment, the induced transcription levels of the selected *Hsf*, *Hsp60*, *Hsp70*, and *Hsp100* genes were low; however, the *sHsps* maintained their induced transcription levels. These gene expression patterns were consistent with the RNA-seq data (Figure 6). Interestingly, in the 2-h plant recovery period after the 45°C treatment, the increased transcription levels of almost all of the selected *Hsf* and *Hsp* genes did not decline (Figure 8).

### *Populu*s Hsfs transcriptionally regulate the expression of different sets of *Hsp* genes

**Figure 9:**
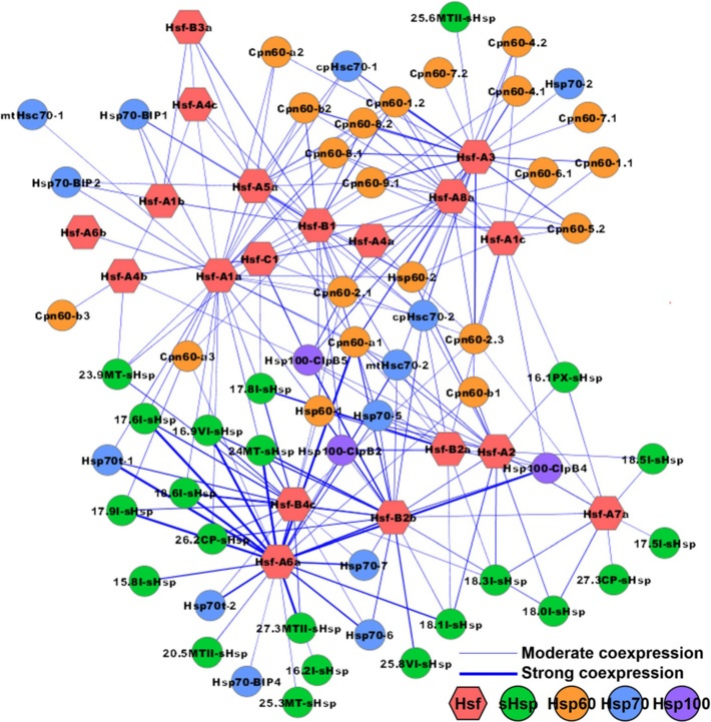
**Coexpression network of** ***Hsfs*** **and** ***Hsps*** **in** ***Populus.*** Nodes represent *Hsfs* and *Hsps* in *Populus*, edges indicate pairwise correlation constructed by WGCNA. Node color codes represent different gene families. Red hexagons indicate *Hsfs*, solid circles with green, orange, blue, and purple indicate *sHsps*, *Hsp60s*, *Hsp70s*, and *Hsp100s*, respectively. Thin edges indicate moderate coexpressions and thick edges indicate strong coexpressions between the two nodes. The network was created using Cytoscape (see Methods).

**Figure 10:**
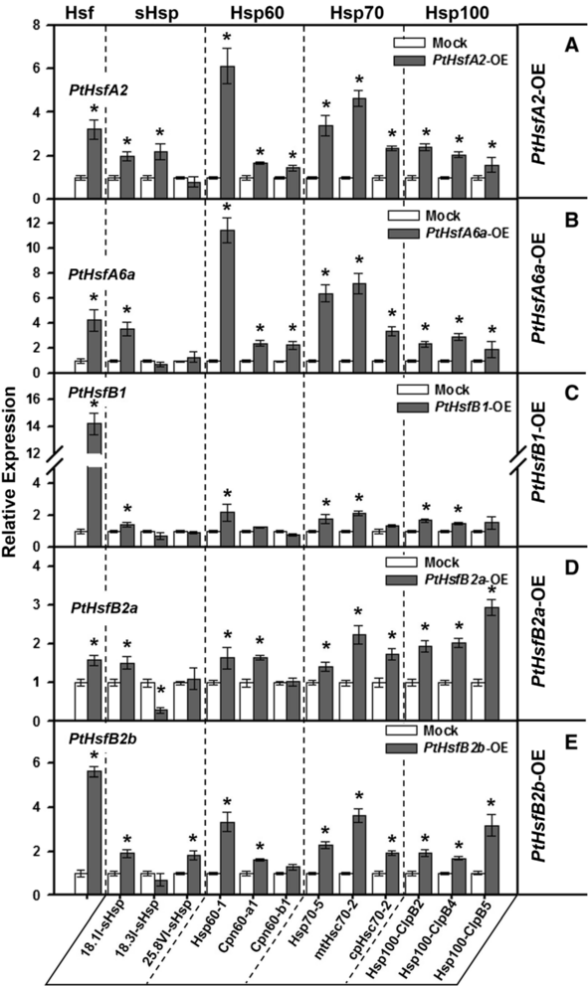
**qRT-PCR validation of transcriptional expression of** ***Hsps*** **regulated by transient overexpression of** ***PtHsfs*** **.** The relative mRNA abundance of selected three *sHsps*, three *Hsp60s*, three *Hsp70s* and three *Hsp100s* were quantified in transient overexpression leaves of *PtHsfA2* **(A)**, *PtHsfA6b* **(B)**, *PtHsfB1* **(C)**, *PtHsfB2a* **(D)**, and *PtHsfB2b* **(E)**. The relative expressions represent log2 expression values. Asterisk, significant differences (*P* < 0.05) between overexpression and mock control.

In *Arabidopsis*, some Hsfs have been shown to promote rapid *Hsp* expression by binding to *cis-*acting sequences, designated as heat shock elements (HSEs), in the promoter of *Hsps* [21,48]. To explore the potential regulatory network between *PtHsfs* and their downstream *PtHsps*, we constructed a coexpression network between *PtHsfs* and *PtHsps* using WGCNA [49]. As shown in Figure 9, there are many coexpression relationships between *Populus Hsfs* and *Hsps*, and one *Hsf* could be coexpressed with several *Hsps*. Moreover, several *PtHsfs*, including *PtHsfA2*, *PtHsfA6a*, and *PtHsfB2a*, showed high coexpression levels when bound to *PtHsps*. To validate the possible regulatory roles of *PtHsfs*, we transiently overexpressed *PtHsfs* in *P. trichocarpa* to detect the transcriptional regulation of the downstream *PtHsps* using qRT-PCR. Two type A *Hsfs* (*PtHsfA2* and *PtHsfA6a*) and three type B *Hsfs* (*PtHsfB1*, *PtHsfB2a*, and *PtHsfB2b*) were selected for infiltration (see Methods). Three days after the infiltration of *PtHsfs*, the expression levels of selected *Hsps* (three *sHsp*, three *Hsp60*, three *Hsp70*, and three *Hsp100*) were examined to evaluate the transcriptional regulatory relationships between selected *PtHsf* and selected *PtHsps* (Figure 10).We revealed that, the expression levels of all of the detected *Hsps*, except *25.8VI-sHsp*, were induced by the overexpression of *PtHsfA2* (Figure 10A). One *sHsps* (*18.1I-sHsp*), two *Hsp60s* (*Hsp60-1* and *Cpn60-a1*), three *Hsp70s* (*Hsp70-5*, *mtHsc70-2*, and *cpHsc70-2*), and two *Hsp100s* (*Hsp100-ClpB2* and *Hsp100-ClpB4*) were significantly induced by the transient overexpression of *PtHsfA6a*, *PtHsfB2a*, and *PtHsfB2b* (Figure 10B, D, and E). However, only a few selected *Hsps* were induced by the overexpression of *PtHsfB1* (Figure 10C).

## Discussion

### Duplications play major roles in the diversification of the *Populus Hsf* and *Hsp* gene families

Gene duplications represent the major mechanism for gene family expansion through either WGD or tandem duplications during evolution [50]. Like most gene families, *Hsf* and *Hsp* appear to have undergone complicated evolutionary processes [11,13]. The abundance of *Hsf* and *Hsp* genes in *P. trichocarpa* may be the result of multiple gene duplication events, represented by a whole genome duplication following multiple segmental and tandem duplications [36]. However, 44 of the 118 *Hsf* and *Hsp* genes lack copies in the corresponding duplicated blocks in the genome of *P. trichocarpa* (Figure 3). This suggested that dynamic rearrangements or segmental losses may have occurred following the segmental duplication. In plants, the exon/intron diversification of gene family members has played an important role in the evolution of multiple gene families [51]. Only 6 of 42 paralogous pairs of *Hsf* and *Hsp* genes exhibited certain degrees of variation in *P. trichocarpa* (Figures 1 and 2). The differences might be derived from the single intron addition or subtraction events during the structural evolution of the *Hsf* or *Hsp* genes.

In *P. trichocarpa*, ~33.4% of predicted genes originated from whole genome duplication and 15.6% from tandem duplications [36]. Our study indicates that the *Hsf* and *Hsp* gene families possess a higher whole genome duplication ratio (47.5%, 56 of 118 genes) and lower tandem duplication ratio (13.5%, 16 of 118 genes) in *P. trichocarpa*, which are dramatically different from the averages. This high retention rate of whole genome duplications and low retention rate of tandem duplications are also inconsistent with the previous studies on other families [52-56]. The low *K*a/*K*s ratio indicates that all gene pairs, except for two (*PtHsf-A6a/PtHsf-A6b* and *Pt19.9CP-sHsp/Pt26.2CP-sHsp*), might have evolved under the influence of purifying selection.

### *Populus Hsf* and *Hsp* genes are vital players in plant development and in response to abiotic stresses

Plant *Hsf* and *Hsp* genes are implicated in a variety of biological processes [6,12,13,57]. The study on the *Populus Hsf* and *Hsp* gene families showed that these genes were differentially expressed in various analyzed tissues. Most *Hsp60* and *Hsp70* genes showed high expression levels in the upper stem (internode 2 and 3), indicating their putative roles in the primary stem growth of *Populus* (Figure 4). Our qRT-PCR results showed that all selected *Populus Hsf* and *Hsp* genes exhibited diverse expression levels in the five examined tissues (Figure 7), indicating that these *Hsf* and *Hsp* genes often participate in plant development. It is worth noting that the expression of *Pt18.3I-sHsp* was obviously higher in stem than in leaves and roots, indicating that it may have potential roles in stem development.

Hsfs, as transcriptional activators of *Hsps*, cooperated with *Hsps* to form a network responding to various stresses. They play a broad role in the tolerance to multiple environmental stress treatments apart from heat stress [6,35]. The response of Hsfs and Hsps to heat and other abiotic stresses overlaps extensively, indicating that *Hsfs* and *Hsps* are important in the cross-talk of multiple environmental stress response pathways [12,13]. Interestingly, under drought stress, most members of the *Populus Hsf*, *Hsp60*, *Hsp70*, and *Hsp100* families were not regulated, while only members of the *sHsp* family were significantly up-regulated (especially *Populus* C-I *sHsp* genes) in the hybrid poplar (Figure 6). The specific response to drought stress of *Populus* C-I *sHsp* might be related to the member expansion of the *Populus* C-I *sHsp* subfamily, but the mechanisms need to be further examined. The expression of *Hsps* could be down-regulated to their initial levels when plants recovered from high temperatures [37]. However, the *Populus sHsps* remained at a high expression level 2 h after the plants’ recovery from 37°C (Figure 8). This indicated that *Populus sHsps* play an irreplaceable role compared with other *Hsps* (*Hsp60*, *Hsp70*, and *Hsp100* genes) in response to heat and drought stress.

### Complex transcriptional regulatory network between *Populus Hsfs* and *Hsps*

Several *PtHsfs*, including *PtHsfA2*, *PtHsfA6a*, and *PtHsfB2a*, showed high coexpression levels with *PtHsps*, indicating that these *PtHsfs* might be the key regulators among the numerous *PtHsfs*. Nishizawa *et al*. [21] showed that *Arabidopsis HsfA2* is a key regulator in the induction of the defense system under several types of environmental stresses. In our study, the overexpression of *PtHsfA2* induced the expression of 11 of 12 *Hsps* (Figure 10A), indicating that *PtHsfA2* is also an activator of the downstream *Hsps*. The expression of *PtHsfA2* was induced under nitrogen deprivation, drought, and heat stresses (Figures 5 and 6), implying that *PtHsfA2* is a key regulator under various stresses in poplars. A set of *PtHsps* could be simultaneously induced by the transient overexpression of *PtHsfA6a*, *PtHsfB2a*, or *PtHsfB2b*, indicating that these *Hsps* were generally regulated by the three *Hsfs* (Figure 10B, D, and E). Recently, Ikeda *et al*. [58] reported that *HsfB1* and *HspB2b* suppress the general heat shock response under non-heat-stress conditions and in the attenuating period. However, *HsfB1* and *HsfB2b* appear to be necessary for the expression of heat stress-inducible *Hsps* under heat stress conditions, which is necessary for acquired thermotolerance. In this study, most detected *Hsps* could not be activated by the overexpression of *PtHsfB1* (Figure 10C), while they could be activated by overexpressing *PtHsfB2a* or *PtHsfB2b* (Figure 10D and E). These results indicated that *PtHsfB1* is not a direct activator of these *Hsps*. Under heat stress, *PtHsfB1* was also induced dramatically, indicating that although *PtHsfB1* could not regulate the downstream *Hsps* directly, it might be involved in the heat stress response in some indirect manner. Tomato (*Solanum lycopersicum*) HsfB proteins may be coactivators of HsfAs [59]. In *Arabidopsis*, the heat stress response is finely regulated by activation and repression activities of *Hsfs* [58]. The expression of a particular *Populus Hsfs* can induce the expression of a different set of *Hsps*, suggesting a complex transcriptional regulatory network between *Populus* Hsfs and *Hsps*. Additionally, the *Hsps* might also be regulated by both the activation and the repression mechanisms during the heat stress response in *Populus*. However, the precise regulatory mechanisms among *Hsfs* and *Hsps* of woody plants during development and stress responses require further investigation.

## Conclusions

In this study, 118 members of *Populus Hsf* and *Hsp* (including *sHsp*, *Hsp60*, *Hsp70* and *Hsp100*) gene families were identified. A comprehensive analysis of these genes, including phylogeny, chromosomal location, gene structure, expression profiling and heat stress responses, was performed. In the phylogenetic tree, the majority of subfamilies contained members from both *Populus* and *Arabidopsis*, which suggested that the functions of most of *Hsfs* and *Hsps* were conserved during evolution. In addition, a gene duplication analysis revealed that the class I cytosolic *sHsp* subfamily had expanded more than that of *Arabidopsis*, showing both the conservation and divergence of gene families during the evolution. An extensive expression analysis indicated that *Populus Hsf* and *Hsp* genes may play various conserved roles in different biological processes in plants. Moreover, the coexpression network and transient overexpression of *PtHsfs* imply that there is a complex transcriptional regulatory network between *Populus Hsfs* and *Hsps*. The present study established a solid foundation for functional research on the *Populus Hsf* and *Hsp* gene families and has improved our understanding of the functions of *Hsf* and *Hsp* genes in woody plants.

## Methods

### Sequence retrieval and phylogenetic reconstruction

Published *Arabidopsis Hsf* and *Hsp* sequences [13] were retrieved and were used as queries in BLAST searches against the *Populus trichocarpa* genome database (http://phytozome.jgi.doe.gov/pz/portal.html#!info?alias=Org_Ptrichocarpa, release 3.0) to identify potential *Populus Hsfs* and *Hsps*. WoLF PSORT (http://psort.hgc.jp/) was used to predict their protein subcellular localizations [60]. The isoelectric points and molecular weights were estimated using the Compute pI/Mw tool from ExPASy (http://web.expasy.org/compute_pi). The phylogenetic trees were constructed using the neighbor-joining method [61] in the MEGA package V5.1 [62] with bootstrap values from 1,000 replicates indicated at each node. To predict putative orthologous genes, a reciprocal Best Blast Hit search was used. The gene names of *Populus Hsfs* are according to Scharf et al. [63] and the gene names of *Populus sHsps* were revised according to their molecular weights in the *P. trichocarpa* genome V3.0 based on Water et al. [64].

### Chromosomal locations, gene structures, and a conserved motif analysis

The chromosomal locations of the *Hsf* and *Hsp* genes were determined using the *Populus* genome browser (http://www.phytozome.net/poplar). Homologous chromosome segments resulting from whole-genome duplication events were identified as described previously [37]. The exon and intron structures were illustrated using Gene Structure Display Server (GSDS, http://gsds.cbi.pku.edu.cn) [65] by aligning the cDNA sequences with the corresponding genomic DNA sequences from Phytozome (http://www.phytozome.net). The conserved motifs in the encoded proteins were analyzed by MEME online program (http://meme.sdsc.edu, v4.9.0) [38]. MEME was run locally with the following parameters: number of repetitions = any, maximum number of motifs = 20, and optimum motif width = 30 to 70 residues for Hsf, Hsp60, Hsp70 and Hsp100. Because of the short sHsp protein length, we set an optimum motif width of between 10 and 40.

### Publicly available microarray data analyses and coexpression network generation

For abiotic and hormonal treatments, the Affymetrix microarray data available in the NCBI GEO database under the series accession numbers GSE26199 (heat stress), GSE17230 (drought stress), and GSE16786 were analyzed [47,66,67]. GSE16786 is composed of the following five subsets: GSE14893 (nitrogen limitation, genotype 1979), GSE14515 (nitrogen limitation, genotype 3200), GSE16783 (1 week after leaf wounding), GSE16785 (90 h after leaf wounding), and GSE16773 (methyl jasmonate-elicited suspension cell cultures). Probe sets corresponding to *Populus Hsf* and *Hsp* genes were identified using the online Probe Match tool POParray (http://aspendb.uga.edu/poparray). For genes with more than one probe sets, the median of expression values was considered. The expression data were gene-wise normalized. The data was normalized using the Gene Chip Robust Multiarray Analysis (GCRMA) algorithm followed by log transformation and average calculations. After normalization and log transformation of data for all the *Populus* genes present on the chip, the log signal intensity values for *Populus* probe set IDs corresponding to *Hsf* and *Hsp* genes were extracted for further analyses. The expression profiles were performed in MultiExperiment Viewer (MeV) v4.7.4 [68].

The WGCNA package [49] provides a robust set of R functions for constructing weighted coexpression networks. The data from the microarray data and our RNA-seq data in Figures 4, 5, 6 were used for the coexpression analysis. The similarity matrix was transformed into an adjacency matrix using a method that employs a power function. The network picture was created using Cytoscape [69].

### Plant material and growth conditions

One-year-old *P. trichocarpa* and hybrid poplar (*P. alba* × *P. glandulosa*) clones (84 K) were grown in a growth chamber under long-day conditions (16 h light/8 h dark) at 23°C. Plant materials for the qRT-PCR of different tissues and under heat treatment conditions were collected from 84 K. For the heat treatment used in RNA-seq, 1-year-old seedlings of *P. alba* × *P. glandulosa* were treated at 37°C for 8 h (10:00 a.m.-18:00 p.m.) and then recovered to 23°C. During the heat treatment, three time points (0 h, 2 h, and R2 h) were selected to sample collection for RNA-seq. At each time point, the 5th internodes of seedlings under both heat treatment and control conditions were collected separately. For the heat treatment used in the qRT-PCR, the chamber was heated to 37°C for 3 h (pretreatment), returned to 23°C for 2 h, heated to 45°C for 3 h (treatment), and then allowed to recover for 2 h. Leaves from three different plants were harvested at seven selected time points during heat stress treatments. For the transient overexpression of *35S::PtHsfs*, *P. trichocarpa* leaves were infiltrated by *Agrobacterium tumefaciens*. Samples were harvested, frozen immediately in liquid nitrogen, and stored at −80°C for further analysis. Three biological replicates were performed.

### Plasmid construction and transient overexpression in *P. trichocarpa*

The coding sequences of *PtHsfA2*, *A6a*, *B1*, *B2a*, and *B2b* were amplified from the cDNA of *P. trichocarpa*, and inserted into pMDC32 to produce the *35S::PtHsfs* constructs using the Gateway cloning system (Invitrogen, Carlsbad, USA). Each *35S::PtHsfs* construct was introduced into the *A. tumefaciens* GV3101 by electroporation. A sample of 1 mL from an overnight *A. tumefaciens* culture was centrifuged at 6,000 × g for 1 min, the supernatant was discarded and the pellet was resuspended in infiltration media (10 mM MgCl_2_ and 150 μM acetosyringone) (OD_600_ = 0.1). Then, the *A. tumefaciens* was infiltrated into *P. trichocarpa* epidermal cells. After 3 days, the infiltrated leaf regions were collected for RNA extraction.

### RNA isolation and real-time qRT-PCR

Total RNA was extracted using the RNeasy Plant Mini Kit (Qiagen, Venlo, Netherlands) with an on-column RNase-free DNase I (Qiagen, Venlo, Netherlands) treatment to remove any contamination of genomic DNA. The first-strand cDNA synthesis was carried out with ~1 μg RNA using the SuperScript III reverse transcription kit (Invitrogen, Carlsbad, USA) and random primers according to the manufacturer’s procedure. Primers with melting temperatures of 58–60°C and amplicon lengths of 150–250 bp were designed using Primer3 software (http://frodo.wi.mit.edu/primer3/input.htm). All primer sequences are listed in Additional file 11: Table S10. Real-time qRT-PCR was conducted on a 7500 Real Time PCR System (Applied Biosystems, CA, USA) using a SYBR Premix Ex Taq™ Kit (TaKaRa, Dalian, China) according to the manufacturer’s instructions. Relative expression levels were calculated using the 2^-ΔΔCt^ method [70]. The *PtActin* and *PtTubulin* genes were used as internal controls.

### Statistical analysis

The statistical significance of differences in measured parameters was tested using the procedures of DPS (Zhejiang University, China). Differences between the means among different tissues or gene pairs were compared using Duncan’s test and Fisher’s protected least significant difference (LSD) test at 0.05 probability levels.

## Additional files

Additional file 1: Table S1.
*Hsf* and *Hsp* gene families in *Populus.*
Additional file 2: Table S2. List of all *Hsf*, *sHsp*, *Hsp60*, *Hsp70* and *Hsp100* gene sequences identified in *Populus*. The complete coding sequences and the corresponding amino acid sequences of *Hsf* and *Hsp* genes identified from *P. trichocarpa* were obtained from Phytozome (http://www.phytozome.net/poplar, release 3.0). Additional file 3: Table S3. Divergence between paralogous *Hsf* and *Hsp* gene pairs in *Populus*. Additional file 4: Table S4. Sequence logos for the conserved motifs of Hsf proteins in *Arabidopsis* and *Populus*. Additional file 5: Table S5. Sequence logos for the conserved motifs of sHsp proteins in *Arabidopsis* and *Populus*. Additional file 6: Table S6. Sequence logos for the conserved motifs of Hsp60 proteins in *Arabidopsis* and *Populus*. Additional file 7: Table S7. Sequence logos for the conserved motifs of Hsp70 proteins in *Arabidopsis* and *Populus*. Additional file 8: Table S8. Sequence logos for the conserved motifs of Hsp100 proteins in *Arabidopsis* and *Populus*. Additional file 9: Figure S1. In silico EST analysis of *Populus Hsfs* and *Hsps*. EST frequency for each gene was calculated by evaluating its EST representation among 17 cDNA libraries available at PopGeneIE (http://popgenie.org/) [43]. Color bar at bottom represents the frequencies of EST counts. P: petioles, IS: imbibed seeds, B: bark, AC: active cambium, FB: flower buds, TW: tension wood, MC: male catkins, DC: dormant cambium, AS: apical shoot, DB: dormant buds, FC: female catkins, CZ: cambial zone, CSL: cold stressed leaves, SL: senescing leaves, YL: young leaves, R: roots, SM: shoot meristem. Additional file 10: Table S9. FPKM of *Populus Hsfs* and *Hsps* during heat treatment obtained from RNA-seq data. One-year-old seedlings of *P. alba* × *P. glandulosa* were treated at 37°C for 8 h (10:00 a.m. ~ 18:00 p.m.) and then recovered to 23°C. Control plants (CK) were grown under 23°C. During the heat treatment, materials collected at three time points were used for RNA-seq. 0 h, before heat treatment; 2 h, 2 h after 37°C heat treatment; R2 h, 2 h after recovered from 37°C to 23°C. At the time points of 2 h and R2 h, the 5th internode of four independent plants in heat treatment group and control group were collected for RNA extraction and RNA-seq. Differential expression was calculated by Cuffdiff using the FPKM.) Additional file 11: Table S10. Primer sequences used in qRT-PCR analysis.
