# Supplementary material for: Hsf and Hsp gene families in Populus: genome-wide identification, organization and correlated expression during development and in stress responses
Source: BMC Genomics. 2015 Mar 14;16(1):181. doi: 10.1186/s12864-015-1398-3 (PMC4373061; doi:10.1186/s12864-015-1398-3)
Supplement: Additional file 3: Table S3. — Divergence between paralogous Hsf and Hsp gene pairs in Populus. [file 12864_2015_1398_MOESM3_ESM.docx]

### Table S3. Divergence between paralogous *Hsf* and *Hsp* gene pairs in *Populus*.

| **No.** | **Gene 1** | **Gene 2** | ***K*a** | ***K*s** | ***K*a/*K*s** | **Duplication** |
| --- | --- | --- | --- | --- | --- | --- |
| 1 | *PtHsf-A1c* | *PtHsf-A1a* | 0.0980 | 0.2767 | 0.3543 | W |
| 2 | *PtHsf-A4c* | *PtHsf-A4a* | 0.0634 | 0.2364 | 0.2683 | W |
| 3 | *PtHsf-A5a* | *PtHsf-A5b* | 0.0630 | 0.2345 | 0.2685 | O |
| 4 | *PtHsf-A6a* | *PtHsf-A6b* | 0.0766 | 0.1432 | 0.5348 | W |
| 5 | *PtHsf-A7a* | *PtHsf-A7b* | 0.0651 | 0.2489 | 0.2614 | W |
| 6 | *PtHsf-A8a* | *PtHsf-A8b* | 0.0867 | 0.3341 | 0.2595 | W |
| 7 | *PtHsf-B2a* | *PtHsf-B2c* | 0.1353 | 0.3547 | 0.3816 | W |
| 8 | *PtHsf-B3a* | *PtHsf-B3b* | 0.0372 | 0.2350 | 0.1584 | W |
| 9 | *PtHsf-B4b* | *PtHsf-B4d* | 0.0551 | 0.3140 | 0.1755 | W |
| 10 | *PtHsf-B4a* | *PtHsf-B4c* | 0.0219 | 0.3212 | 0.0681 | W |
| 11 | *Pt12.2I-sHsp* | *Pt13.1I-sHsp* | 0.2195 | 0.9674 | 0.2269 | O |
| 12 | *Pt14.5I-sHsp* | *Pt17.9I-sHsp* | 0.1025 | 0.2806 | 0.3655 | W |
| 13 | *Pt16.2I-sHsp* | *Pt17.6I-sHsp* | 0.3476 | 1.6861 | 0.2061 | O |
| 14 | *Pt16.9VI-sHsp* | *Pt25.8VI-sHsp* | 0.0685 | 0.4425 | 0.1548 | W |
| 15 | *Pt17.8I-sHsp* | *Pt19.0I-sHsp* | 0.0395 | 0.1770 | 0.2234 | T |
| 16 | *Pt18.1I-sHsp* | *Pt18.2I-sHsp* | 0.0472 | 0.4931 | 0.0957 | W |
| 17 | *Pt18.3I-sHsp* | *Pt18.6I-sHsp* | 0.0532 | 0.3164 | 0.1683 | W |
| 18 | *Pt19.9CP-sHsp* | *Pt26.2CP-sHsp* | 0.2030 | 0.3845 | 0.5280 | W |
| 19 | *Pt22.4V-sHsp* | *Pt23.1V-sHsp* | 0.0728 | 0.3537 | 0.2058 | W |
| 20 | *PtHsp60-1* | *PtHsp60-2* | 0.0193 | 0.2026 | 0.0953 | W |
| 21 | *PtCpn60-1.1* | *PtCpn60-1.2* | 0.0253 | 0.2445 | 0.1035 | W |
| 22 | *PtCpn60-2.1* | *PtCpn60-2.3* | 0.0165 | 0.2636 | 0.0625 | O |
| 23 | *PtCpn60-4.1* | *PtCpn60-4.3* | 0.0110 | 0.2116 | 0.0519 | W |
| 24 | *PtCpn60-5.1* | *PtCpn60-5.2* | 0.0220 | 0.3177 | 0.0691 | O |
| 25 | *PtCpn60-6.1* | *PtCpn60-6.2* | 0.0142 | 0.2481 | 0.0572 | W |
| 26 | *PtCpn60-7.1* | *PtCpn60-7.2* | 0.0214 | 0.4218 | 0.0506 | O |
| 27 | *PtCpn60-8.1* | *PtCpn60-8.2* | 0.0260 | 0.3239 | 0.0803 | W |
| 28 | *PtCpn60-9.1* | *PtCpn60-9.2* | 0.0155 | 0.2775 | 0.0559 | W |
| 29 | *PtCpn60-a1* | *PtCpn60-a2* | 0.0247 | 0.2534 | 0.0974 | W |
| 30 | *PtCpn60-a3* | *PtCpn60-a4* | 0.0093 | 0.0342 | 0.2708 | T |
| 31 | *PtCpn60-b1* | *PtCpn60-b2* | 0.0154 | 0.2442 | 0.0632 | W |
| 32 | *PtHsp70-1* | *PtHsp70-5* | 0.0252 | 0.6224 | 0.0405 | T |
| 33 | *PtHsp70-2* | *PtHsp70-6* | 0.0222 | 0.2930 | 0.0759 | T |
| 34 | *PtHsp70-3* | *PtHsp70-4* | 0.0154 | 0.4375 | 0.0353 | W |
| 35 | *PtHsp70-7* | *PtHsp70-9* | 0.0501 | 0.7145 | 0.0701 | T |
| 36 | *PtHsp70-9* | *PtHsp70-8* | 0.0545 | 0.3047 | 0.1789 | W |
| 37 | *PtHsp70-BIP1* | *PtHsp70-BIP2* | 0.0120 | 0.3354 | 0.0359 | W |
| 38 | *PtmtHsc70-1* | *PtmtHsc70-2* | 0.0264 | 0.2940 | 0.0899 | W |
| 39 | *PtcpHsc70-1* | *PtcpHsc70-2* | 0.0184 | 0.2575 | 0.0714 | O |
| 40 | *PtHsp70t-1* | *PtHsp70t-2* | 0.0352 | 0.2434 | 0.1448 | W |
| 41 | *PtHsp100-ClpB1* | *PtHsp100-ClpB2* | 0.0010 | 0.0076 | 0.1259 | T |
| 42 | *PtHsp100-ClpB3* | *PtHsp100-ClpB4* | 0.0338 | 0.2277 | 0.1484 | O |
| Gene pairs were identified at the terminal nodes of the phylogenetic tree shown in Fig. 1 and Fig. 2. Synonymous (*K*s) and nonsynonymous substitution (*K*a) rates are presented for each pair. Gene pairs were generated by tandem duplication (T), whole genome duplication (W), or other (O) events are indicated in the table. | | | | | | |
|  |  |  |  |  |  |  |
|  |  |  |  |  |  |  |
|  |  |  |  |  |  |  |
|  |  |  |  |  |  |  |
