# Supplementary material for: Hsf and Hsp gene families in Populus: genome-wide identification, organization and correlated expression during development and in stress responses
Source: BMC Genomics. 2015 Mar 14;16(1):181. doi: 10.1186/s12864-015-1398-3 (PMC4373061; doi:10.1186/s12864-015-1398-3)
Supplement: Additional file 4: Table S4. — Sequence logos for the conserved motifs of Hsf proteins in Arabidopsis and Populus. [file 12864_2015_1398_MOESM4_ESM.docx]

**Table S4. Sequence logos for the conserved motifs of Hsf proteins in *Arabidopsis* and *Populus*.**

**Hsf motif**

**Motif 1**

E-value 5.5e-2296

Width 59

Sites 48


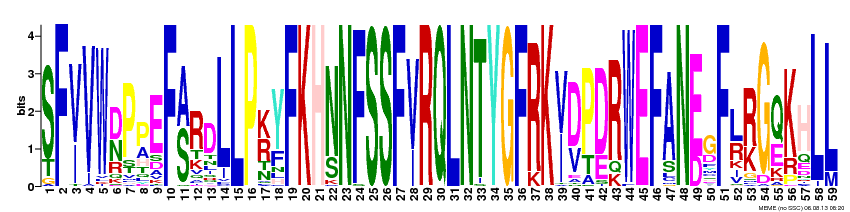


SF[VI]VW[DN]PPEF[AS]RDLLP[KR]YFKHNNFSSF[VI]RQLNTYGFRK[VI][DV]P[DE]RWEFAN[ED]GF[LR][RK]G[QE]KHLL

## Motif 2

E-value 2.6e-819

Width 30

Sites 48


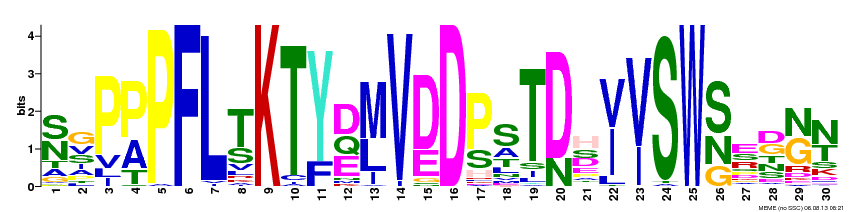


[SN]GP[PA]PFL[TS]KTY[DEQ][ML]V[DE]D[PS]ST[DN]H[VI][VI]SW[SNG]E[DG][NG][NT]

## Motif 3

E-value 1.7e-749

Width 59

Sites 34


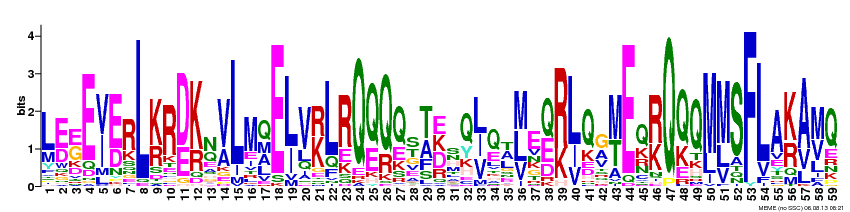


L[ED][EG]E[VI][ED]RL[KR]R[DE][KR][NQ][VA]LMQE[LI]V[KR]LRQ[QE]QQS[TA][EDK]xQ[LIV]Q[AT][MLV]E[QE][RK][LI]QG[MT]EQ[RK]Q[QK]QM[ML]SFLA[KR][AV][MV]Q

## Motif 4

E-value 1.0e-187

Width 49

Sites 15


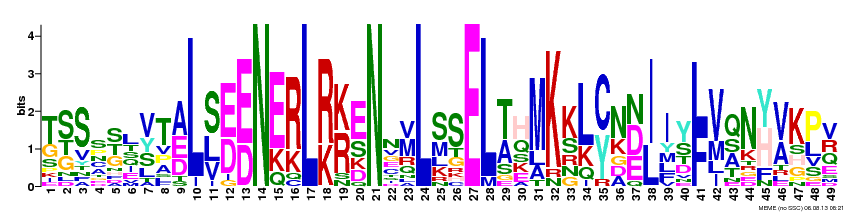


[TG][SGT]SS[SGT]L[VSY]T[AED]L[SLV][ED][ED]N[EK][RK]L[RK][KR][ES]NN[MV]L[SM]SEL[TA][HQ]MK[KS][LK][CY][NK][DNE][IL]I[YS][LF][VM][QAS]N[HY][VA]K[PL][VQR]

## Motif 5

E-value 4.4e-130

Width 40

Sites 32


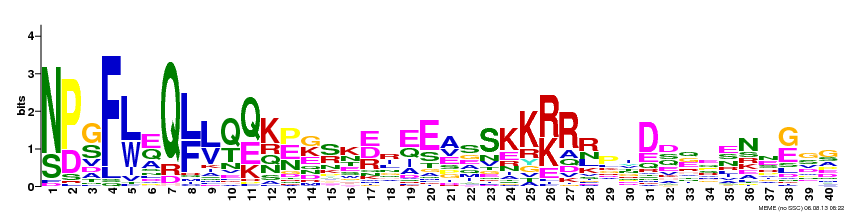


[NS][PD][GS]F[LW][EQ]Q[LF][LV][QT][QE]K[PE]GSK[ED]xEEASSKK[RK]RRPxDxxxxNx[GE]xG

## Motif 6

E-value 2.3e-111

Width 30

Sites 32


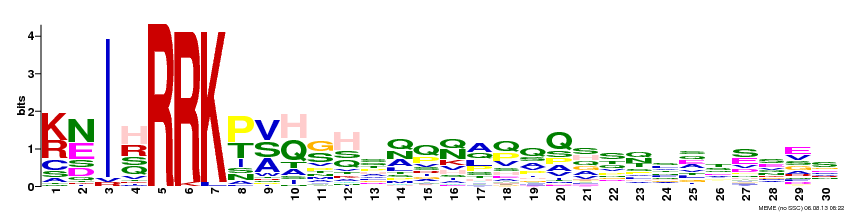


[KR][NE]IHRRK[PT][VSA][HQ][GS]HxQQ[NQ]AQQQxSxxxxSxxS

## Motif 7

E-value 2.8e-043

Width 42

Sites 6


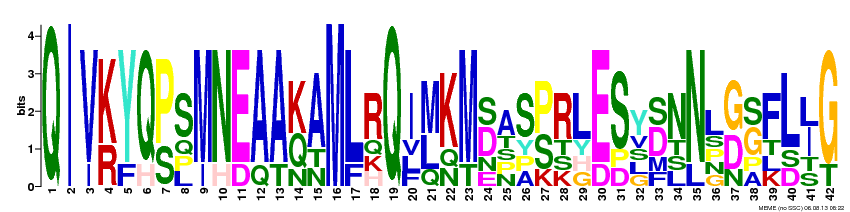


QIV[KR]YQ[PS]SMNEAA[KQ]AMLRQI[ML]KM[DS]AS[PS]RLESY[DS]NNL[GD][GS]FL[IL]G

## Motif 8

E-value 4.3e-043

Width 30

Sites 16


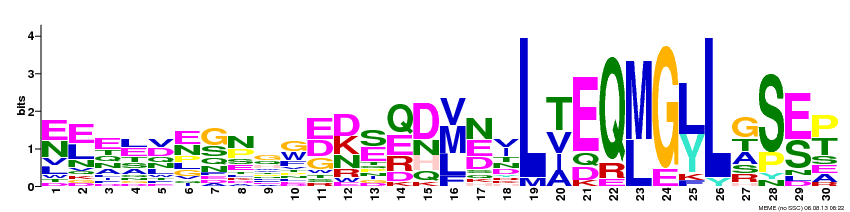


[EN][EL]ExxEGNxG[ED][DK][SE][QE]D[MVL][NE]xL[TV]EQ[ML]G[LY]LGS[ES][PT]

## Motif 9

E-value 2.8e-027

Width 69

Sites 3


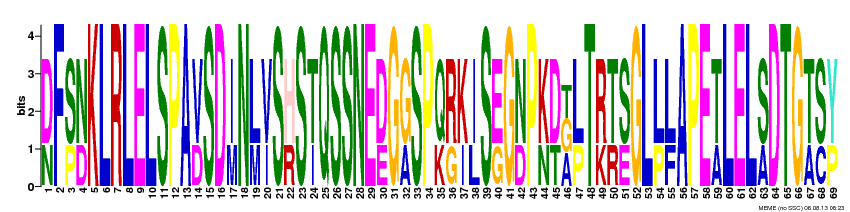


[DN]F[SP][ND]KLRLELSPA[VD]SD[IM]N[LM][VI]S[HR]S[TI]QSSNE[DE]G[GA]SP[QK][RG][KI][IL]S[EG]G[ND]P[KN][DT][AGT][LP]T[RK][TR][SE]GL[LP][LF]APE[TA]LEL[SA]DTG[TA][SC][YP]

## Motif 10

E-value 2.2e-024

Width 30

Sites 12


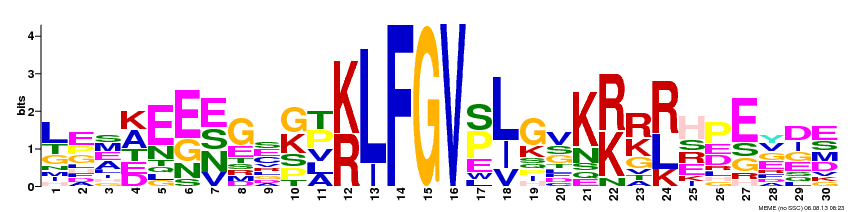


LEx[AK]E[EG][ENS]Gx[GK][PT][KR]LFGV[PS][LI]GVK[RK][RK][RL]HPExDE

## Motif 11

E-value 4.3e-017

Width 70

Sites 4


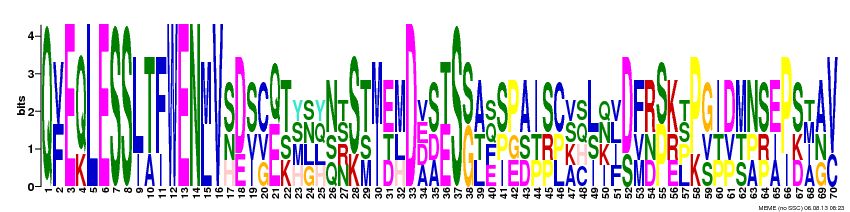


Q[FV]E[QK]LESS[LI][TA][FI]WEN[LM]V[SHN][DE][SIV][CGV][EQ][TKS][HMSY][GLNS][HLQY][NQS][NRST][SK][TMS][IM][EDT][MHL]D[ADEV][SAD][ET]S[GS][ALT][EFQS][SIP][PEG][ADS][IPT][SPR][CLP][AKSV][CHQS][LIS][IKNQ][FILV][DS][FMV][RDN][PS][KER][LPST][PK][GSV][IPT][DPV][MST][NAP][SPR][EAI][PI][SDK][AIMT][AGN][VC]

## Motif 12

E-value 9.2e-016

Width 42

Sites 3


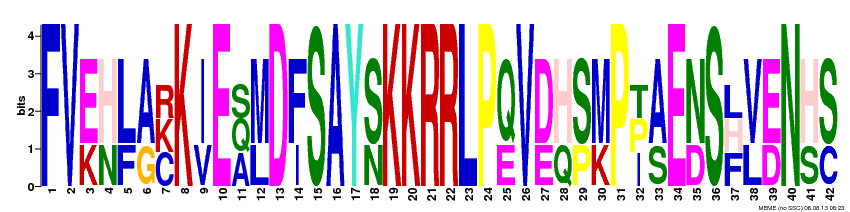


FV[EK][HN][LF][AG][CKR]K[IV]E[AQS][ML]D[FI]SAY[SN]KKRRLP[QE]V[DE][HQ][SP][MK]P[IPT][AS]E[ND]S[FHL][VL][ED]N[HS][SC]

## Motif 13

E-value 1.1e-014

Width 70

Sites 2


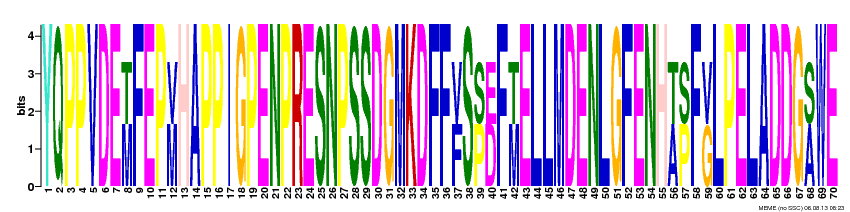


YQPPVDE[MT]FEP[MV]HAPPIGPENPRESNPSSDGMKDFF[FV]S[PS][DE]F[MT]ELLMDENLGFENH[AT][PS]F[GV]LPELADDG[AS]WE

## Motif 14

E-value 2.5e-014

Width 59

Sites 2


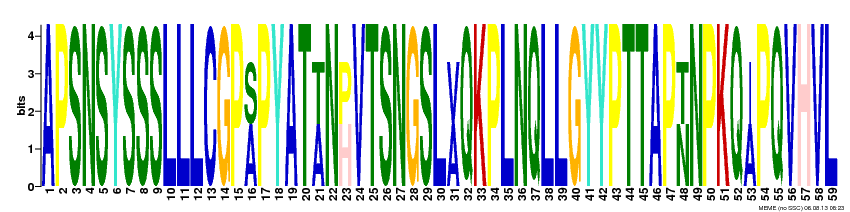


APSNSYSSSLLLCGP[AS]PYAT[AT]N[HP]VTSNGSL[AV]QKPLNQLLGYYPTTAP[NT]NPKQ[AI]PQVHVL

## Motif 15

E-value 2.2e-012

Width 59

Sites 2


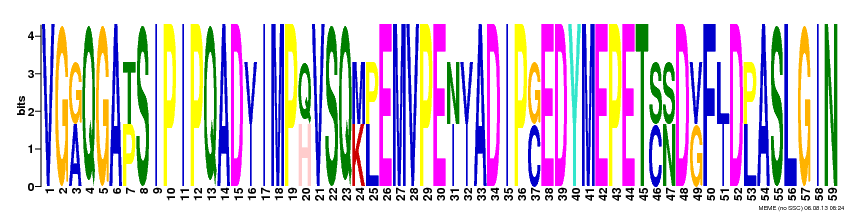


VG[AG]QGA[PT]SIPIPQAD[IV]IMP[HQ]VSQ[KM][LP]EMVPE[IN][IV]ADIP[CG]EDYMEPET[CS][NS]D[GV]F[IL]D[LP]ASLGIN

## Motif 16

E-value 4.6e-011

Width 39

Sites 5


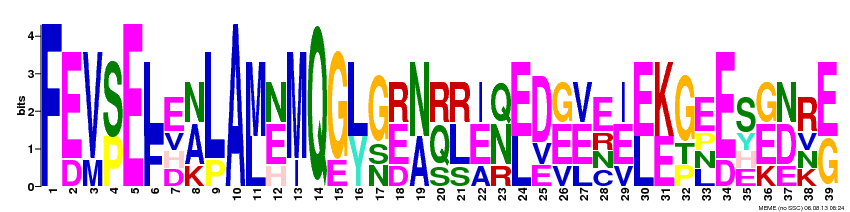


F[ED][VM][SP]E[LF][EDHV][ANK][LP]A[ML][ENH][MI]Q[GE][LY][GNS][ERD][NA][QRS][LRS][EIA][NQR][EL][DEV][EGV][EVL][ECNR][EIV][EL][KE][GPT][ELNP][ED][SEHY][EGK][DNE][RKNV][EG]

## Motif 17

E-value 5.3e-011

Width 48

Sites 3


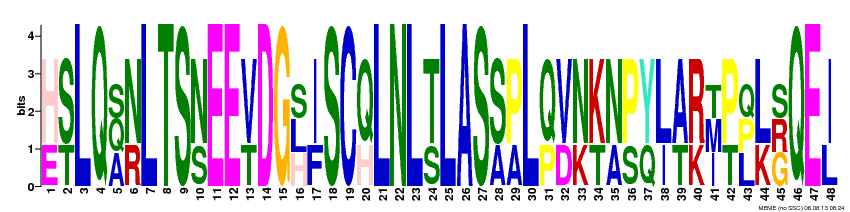


[HE][ST]LQ[AQS][NR]LTS[NS]EE[VT]DG[HLS][IF]SC[QH]LNL[TS]LAS[SA][PA]L[QP][VD][NK][KT][NA][PS][YQ][LI][AT][RK][IMT][PT][LPQ][LK][GRS]QE[IL]

## Motif 18

E-value 3.0e-009

Width 30

Sites 7


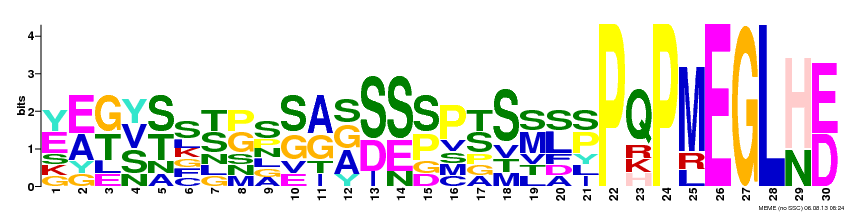


[EY][EA][GT][SVY][ST]S[ST][GP]S[SG][AG][AGS][SD][SE][SP]P[ST]S[MS][LS][PS]PQPMEGL[HN][ED]

## Motif 19

E-value 1.2e-006

Width 35

Sites 2


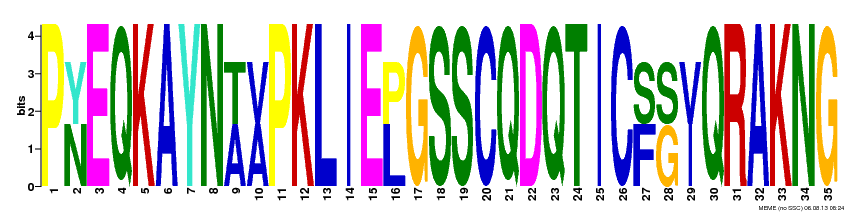


P[NY]EQKAYN[AT][AV]PKLIE[LP]GSSCQDQTIC[FS][GS][IV]QRAKNG

## Motif 20

E-value 3.1e-005

Width 30

Sites 6


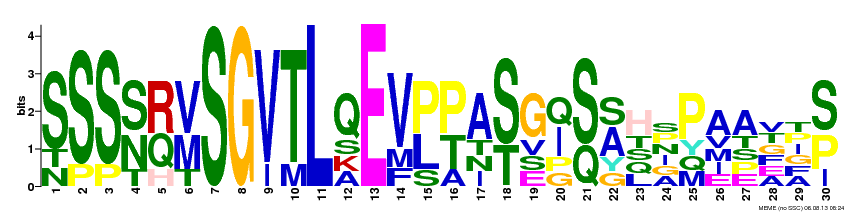


SSS[SN][RQ][VM]SGVTLQEV[PL][PT]A[ST]G[IQ][SQ][AS]HxPAAxx[SP]
