# Supplementary material for: Hsf and Hsp gene families in Populus: genome-wide identification, organization and correlated expression during development and in stress responses
Source: BMC Genomics. 2015 Mar 14;16(1):181. doi: 10.1186/s12864-015-1398-3 (PMC4373061; doi:10.1186/s12864-015-1398-3)
Supplement: Additional file 5: Table S5. — Sequence logos for the conserved motifs of sHsp proteins in Arabidopsis and Populus. [file 12864_2015_1398_MOESM5_ESM.docx]

**Table S5. Sequence logos for the conserved motifs of sHsp proteins in *Arabidopsis* and *Populus*.**

**sHsp Motif**

**Motif 1**

E-value 1.9e-714

Width 30

Sites 50
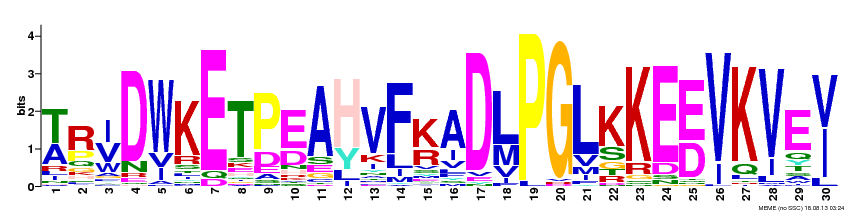


[TA]R[IV]DWKETP[ED]A[HY]VF[KR]AD[LM]PGLKKE[ED]VKVE[VI]

## Motif 2

E-value 6.9e-598

Width 28

Sites 41


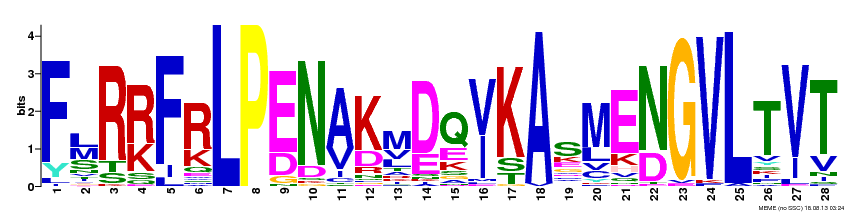


F[LM]R[RK]FRLPEN[AV]KMDQ[VI]KASME[ND]GVLTVT

## Motif 3

E-value 6.3e-376

Width 26

Sites 28


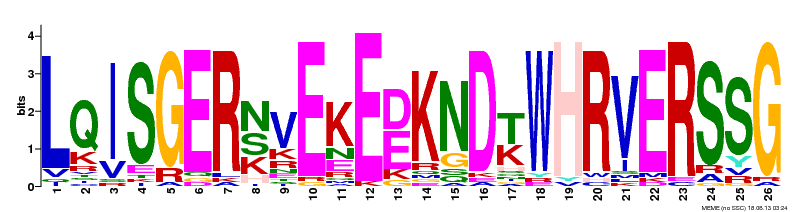


LQISGER[NSK]VEKE[DE]KND[TK]WHRVERSSG

## Motif 4

E-value 9.4e-114

Width 14

Sites 27


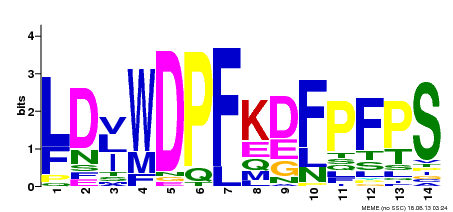


[LF]D[ILV]WDPFK[DE]FPFPS

## Motif 5

E-value 4.3e-103

Width 14

Sites 20


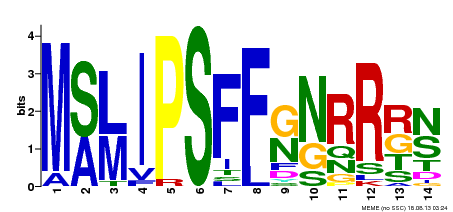


M[SA][LM]IPSFF[GN][NG]RR[RGT][NS]

## Motif 6

E-value 5.7e-082

Width 14

Sites 21


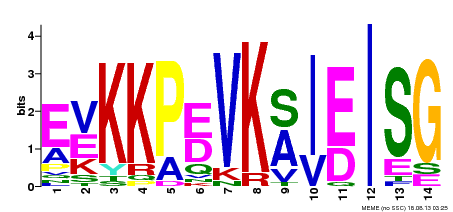


E[VE]KKP[ED]VK[SA][IV][ED]ISG

## Motif 7

E-value 5.7e-057

Width 24

Sites 10


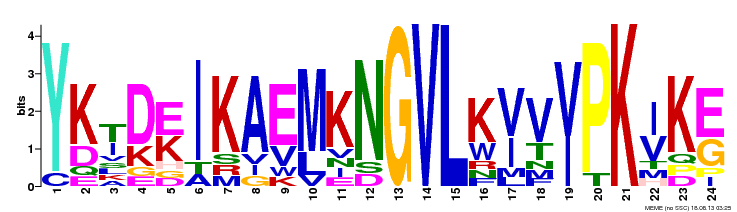


Y[KD][TI][DK][EK]IKA[EV][ML]KNGVL[KW][VI][VT][VI]PK[IV]K[EG]

## Motif 8

E-value 2.0e-044

Width 19

Sites 16


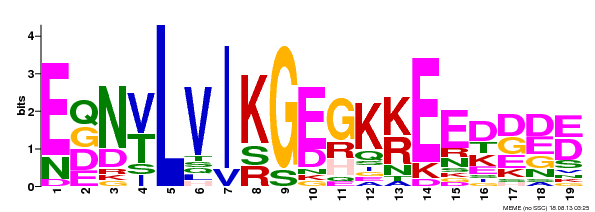


E[QDG]N[VT]LVIKGEGK[KR]EED[DG][DE][DE]

## Motif 9

E-value 1.2e-026

Width 40

Sites 3


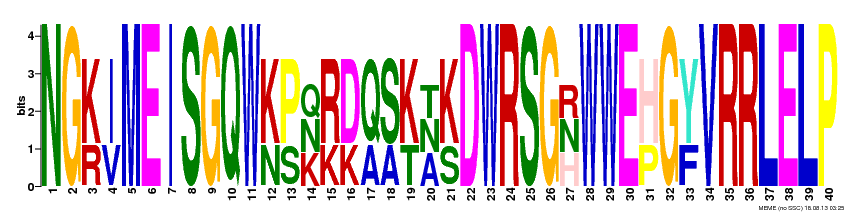


NG[KR][IV]MEISGQW[KN][PS][KNQ][RK][DK][QA][SA][KT][ANT][KS]DWRSG[HNR]WWE[HP]G[YF]VRRLELP

## Motif 10

E-value 7.9e-023

Width 10

Sites 16


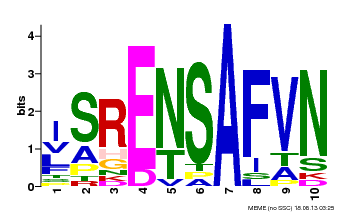


ISRE[NT]SAFVN

## Motif 11

E-value 1.6e-019

Width 36

Sites 3


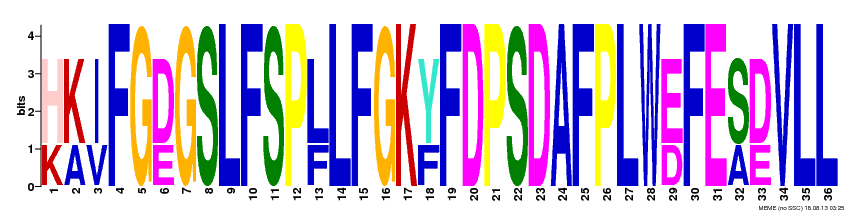


[HK][KA][IV]FG[DE]GSLFSP[LF]LFGK[YF]FDPSDAFPLW[ED]FE[SA][DE]VLL

## Motif 12

E-value 9.1e-014

Width 29

Sites 4


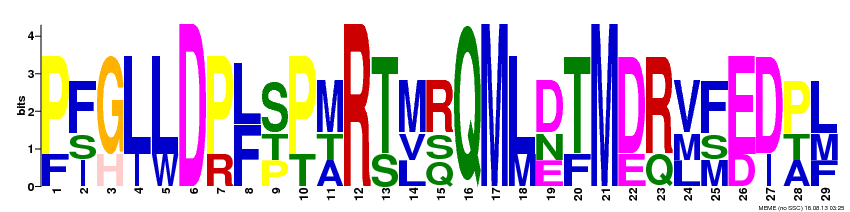


[PF][FIS][GH][LI][LW]D[PR][FL][SPT][PT][MAT]R[TS][MLV][RQS]QM[LM][DEN][TF]M[DE][RQ][VLM][FMS][ED][DI][PAT][LFM]

## Motif 13

E-value 3.0e-012

Width 35

Sites 3


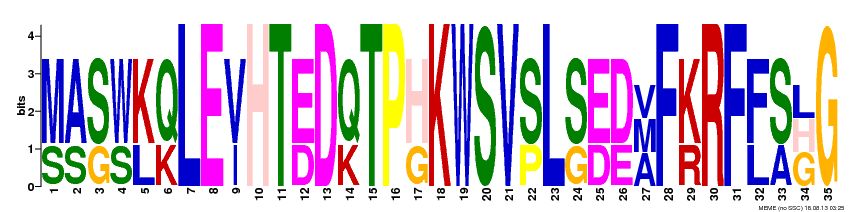


[MS][AS][SG][WS][KL][QK]LE[VI]HT[ED]D[QK]TP[HG]KWSV[SP]L[SG][ED][DE][AMV]F[KR]RF[FL][SA][GHL]G

## Motif 14

E-value 3.1e-008

Width 14

Sites 7


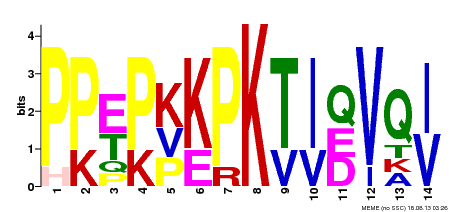


P[PK][ET][PK][KPV][KE]PK[TV][IV][QDE]VQ[IV]

## Motif 15

E-value 1.1e-005

Width 14

Sites 4


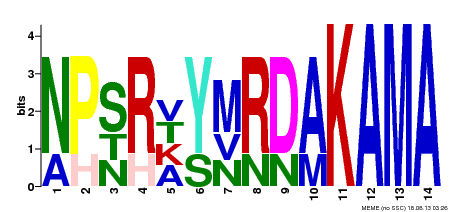


[NA][PH][SNT][RH][AKTV][YS][MNV][RN][DN][AM]KAMA

## Motif 16

E-value 6.6e-005

Width 30

Sites 3


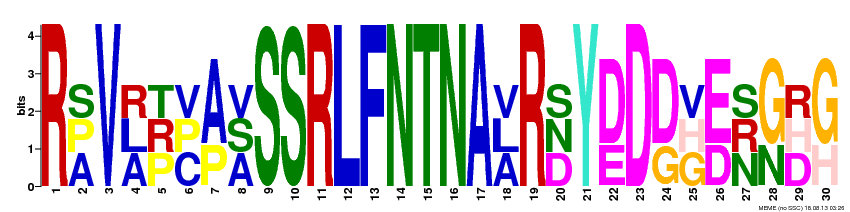


R[APS]V[ALR][PRT][CPV][AP][ASV]SSRLFNTNA[ALV]R[DNS]Y[DE]D[DG][GHV][ED][NRS][GN][DHR][GH]

## Motif 17

E-value 1.1e-003

Width 27

Sites 3


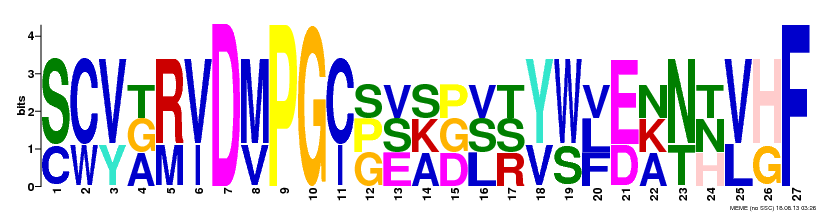


[SC][CW][VY][AGT][RM][VI]D[MV]PG[CI][GPS][ESV][AKS][DGP][LSV][RST][YV][WS][FLV][ED][AKN][NT][HNT][VL][HG]F

## Motif 18

E-value 2.1e-003

Width 26

Sites 2


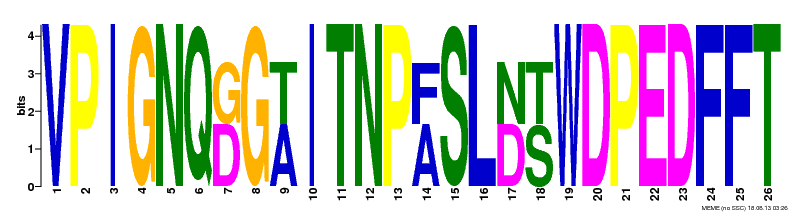


VPIGNQ[DG]G[AT]ITNP[AF]SL[DN][ST]WDPEDFFT

## Motif 19

E-value 2.5e-002

Width 20

Sites 2


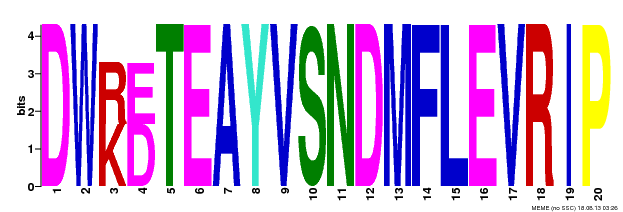


DW[KR][DE]TEAYVSNDMFLEVRIP

## Motif 20

E-value 5.2e-002

Width 36

Sites 2


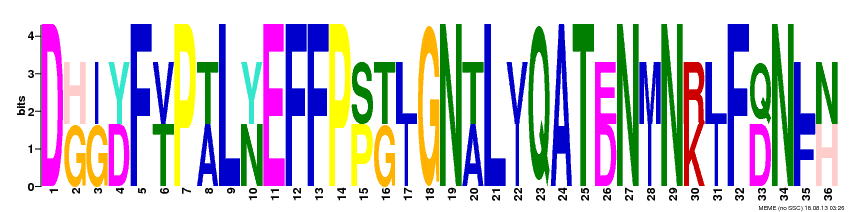


D[GH][GI][DY]F[TV]P[AT]L[NY]EFFP[PS][GT][IL]GN[AT]L[IV]QAT[DE]N[IM]N[KR][IL]F[DQ]N[FL][HN]
