# Supplementary material for: Hsf and Hsp gene families in Populus: genome-wide identification, organization and correlated expression during development and in stress responses
Source: BMC Genomics. 2015 Mar 14;16(1):181. doi: 10.1186/s12864-015-1398-3 (PMC4373061; doi:10.1186/s12864-015-1398-3)
Supplement: Additional file 6: Table S6. — Sequence logos for the conserved motifs of Hsp60 proteins in Arabidopsis and Populus. [file 12864_2015_1398_MOESM6_ESM.docx]

**Table S6. Sequence logos for the conserved motifs of Hsp60 proteins in *Arabidopsis* and *Populus*.**

**Hsp60 Motif**

**Motif 1**

E-value 1.8e-767

Width 57

Sites 40


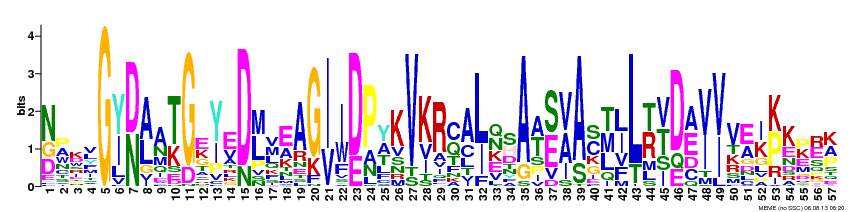


[NDG]PKxG[YI][DN][AL]A[TK]GE[YI]ED[LM]VEAG[IV][IW][DE]P[YA]KVKR[CQ]A[LI][QN]SA[AT][SE][VA]A[CS][TML][LI]L[TR][VT]D[AE][VI][VI]V[EA][IK][KP]K[KP][ER][KA]

## Motif 2

E-value 3.8e-753

Width 43

Sites 45


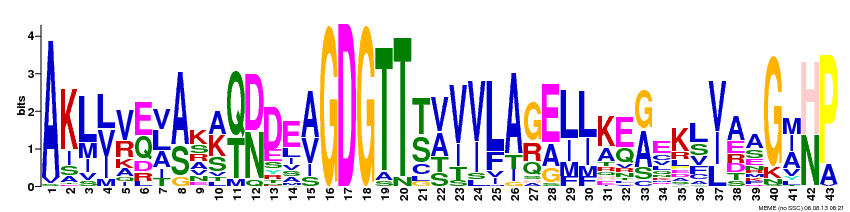


AKL[LVI][VR][EQ][LVA][AS]K[AKS][QT][DN]DE[AV]GDGTT[TS][VA][VI][VI]LA[GR][EA][LI][LI][KA][EQ][GA]EK[LS][VIL]AAG[AIM][HN]P

## Motif 3

E-value 1.2e-821

Width 59

Sites 41


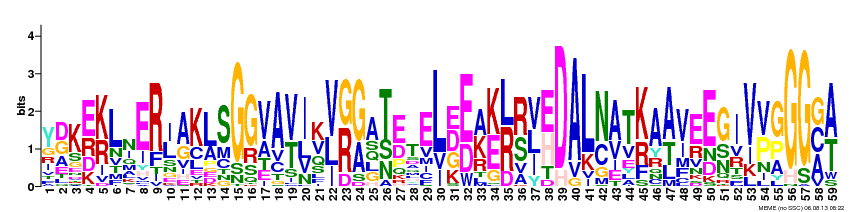


Y[DG]K[ER][KR]LNER[IL]AKLSGGVA[VT]I[KV][VL][GR][GA]A[TS]ETEL[EDG][ED][AK][KE][LR][RS][VLI][EH]DAL[NC][AV]T[KR]A[AT]VEEGIV[VP][GP]GG[GCA][AT]

## Motif 4

E-value 4.6e-697

Width 42

Sites 44


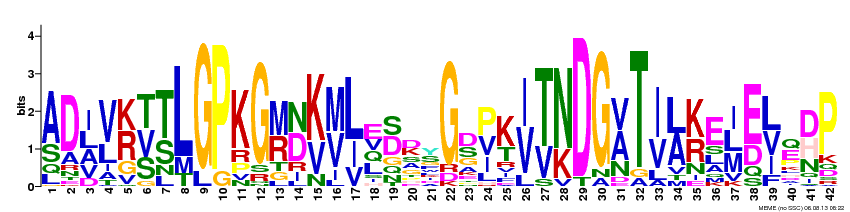


[AS]D[ILA][VL][KR][TVS][TS]LGPKG[MR][ND][KV][MV][LI]E[SD]xYG[DS][PVI][KT][IV][TV][NK]DG[VA]T[IV][LA][KR]E[ILM][ED][LVI][EQ][DH]P

## Motif 5

E-value 2.8e-647

Width 70

Sites 19


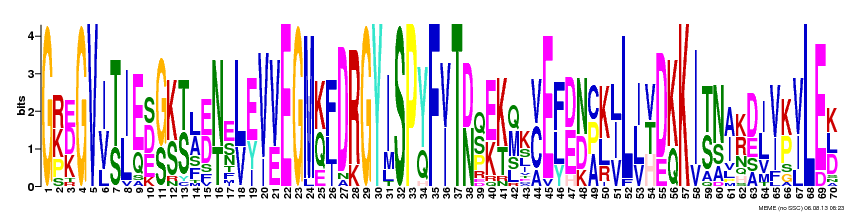


G[RKP][DEK]GV[IV][TS][IL][EQ][DSE][GS][KS][TS][LA][ED][NT][ES][LV][EY][VI][VE]EGM[KQ][FLI]DRGYISP[YQ]F[VI]T[DN][QSP][EK][KT][MQ]K[CV]E[FLY][DE][ND][CAP][KL][ILV]L[ILV][VHT][DE][KQ]KI[TS][NS][AI][KR][DE][IL][VI][KP][VI]LE[KDL]

## Motif 6

E-value 1.2e-631

Width 70

Sites 19


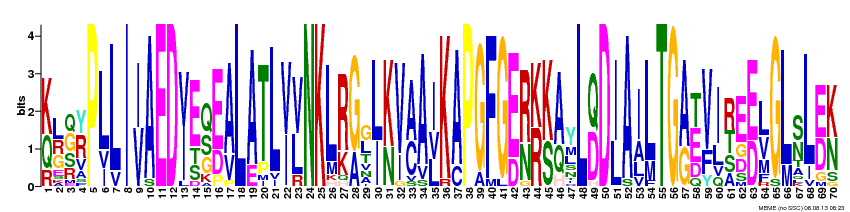


[KQ][LGR][GQS][RY]PLLI[IV]AED[IV]E[QSG][ED][AV]LATL[VI][VL]NKL[RK][GA][GL][LI][KN][VI][AC]A[ILV]KAPGFG[ED][RN][KR][KS][AQ]YL[QD]D[IL]A[IA]LTG[AG][ET][VF][IL][RTS]E[ED][LV]GL[SN]L[ED][KN]

## Motif 7

E-value 6.9e-572

Width 42

Sites 46


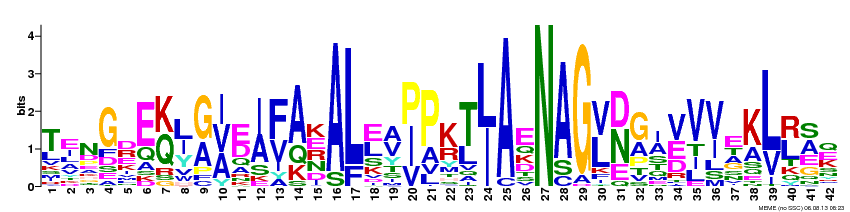


TENGxE[KQ][LI][GA][IVA][ED][IA][FV]AKAL[EL][AV][PI]PK[TL][LI]AENAG[VL][DN]G[AI][VE]V[VI]EK[LV][RL][SA]x

## Motif 8

E-value 1.0e-317

Width 54

Sites 24


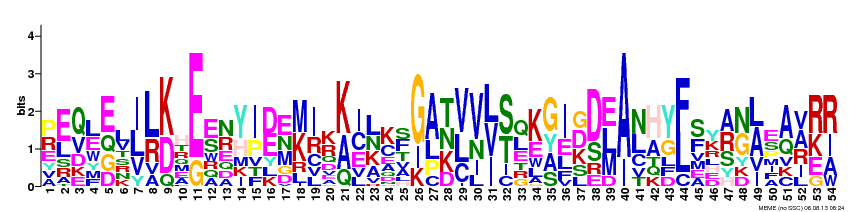


[PR][EL][QV][LE][EGQ]L[ILV][LR][KD]H[EG][ES][NR][YH][IP][DE][EMN][MK][IR][KR][KA][ICE][LKN][KC][FS][GI][AL][TKN][VLC][VN][LVI][ST][QL][KE][GAY][IE][DGK][DS][ELM][AI][NL][HA][YG][FL][SF]Y[ARS][NG][AL]E[AQ][AVR][RKE][RIA]

## Motif 9

E-value 8.0e-287

Width 46

Sites 27


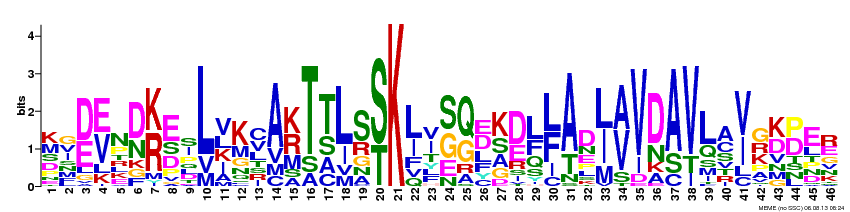


Kx[DE][EV]N[DN][KR]ExLVKCA[KR]T[TS]LS[ST]K[LI][IV][SG][QG][DE][KS][DE][LF][LF]AD[LI][AV]VDAVLA[VI]G[KD][PD]ER

## Motif 10

E-value 4.9e-226

Width 40

Sites 24


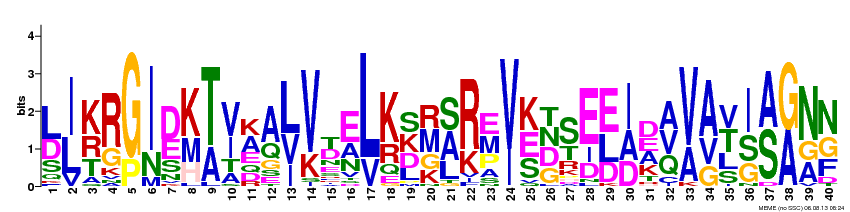


[LD][IL][KRT][RG][GP][IN][DE][KMH][TA][VI][AK][AQ][LVI][VK][TD][EA][LV][KR][SKD][MRG][SAL][RK][EMP][VI][KES][DNTG][ST]E[ELD][IAD][DAE][AVQ][VA][AVG][TVL][ISG][AS][GA][NAG][NGF]

## Motif 11

E-value 1.5e-255

Width 42

Sites 19


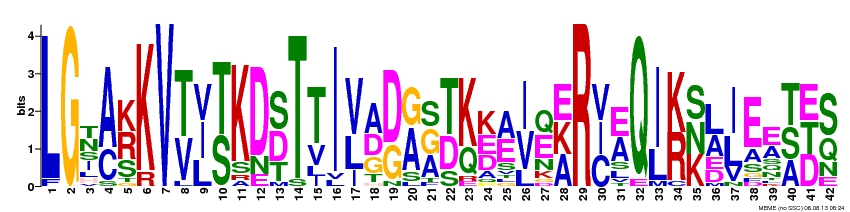


LG[NT][AC][KRS]KV[TV][IVL][TS]KD[SDT]T[TV]I[VL][ADG][DG][AG][GSA][TD][KQ][KED][AE][IV][EQ][EAK]R[VCI][EA]Q[IL][KR][SKN][LA][IL]E[EA][TSA][EDT][SQ]

## Motif 12

E-value 4.8e-209

Width 50

Sites 29


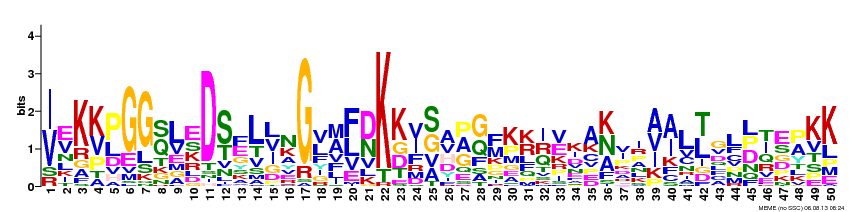


[IV][EV]K[KV][PL]GG[QS]LED[ST][EF]L[LIV]NG[VL][AM][FL][DN]KK[IV][SG]A[APG][GQ][FM]K[KR][IQR][VE]KA[KNA]xx[AVI][AI]L[TL]xL[LDP][IT]E[PA]KK

## Motif 13

E-value 2.0e-099

Width 30

Sites 18


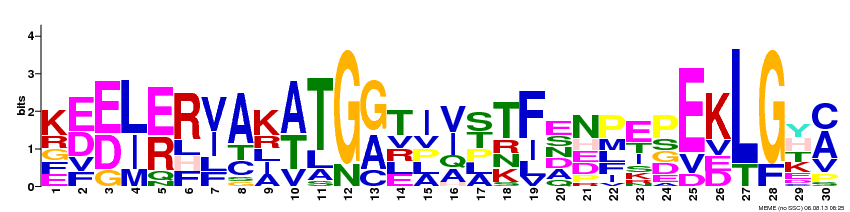


K[ED][ED][LI][ER]R[VI]AK[AT]TG[GA]TI[VI][ST]T[FI]ENPEPEKLG[HY][AC]

## Motif 14

E-value 3.5e-093

Width 42

Sites 11


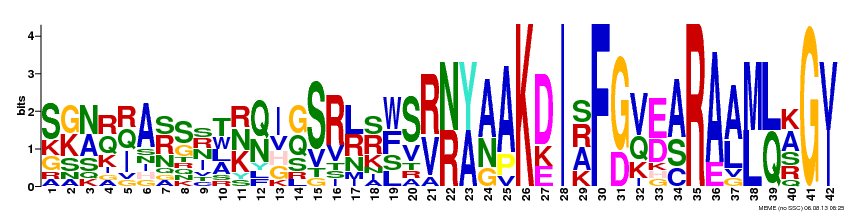


S[GK][NA][QR][IQR]A[RS]SxT[KNR][QN]I[GQ]SR[LR][RS][WF]S[RV][NR][YA][AN]AKDI[ARS]F[GD][VQ][ED][AS]RA[AL][ML][LQ][AK]G[VI]

## Motif 15

E-value 1.5e-094

Width 42

Sites 8


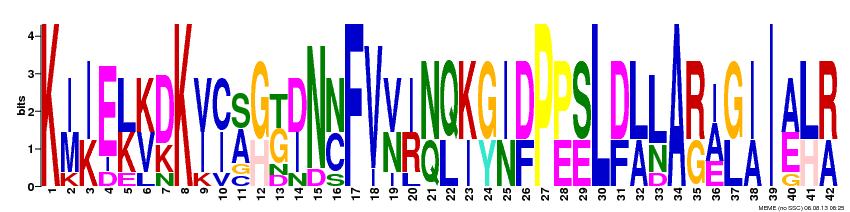


K[IM][IK]E[LK][KV][DK]K[VI][CI][AS][GH][GT][DI]N[NC]FV[VN][IR][NQ][QL][KI][GY][IN][DF]P[PE][SE]L[DF][LA][LN]A[RG][AIE][GL][IA]I[AE][LH][RA]

## Motif 16

E-value 4.7e-074

Width 30

Sites 20


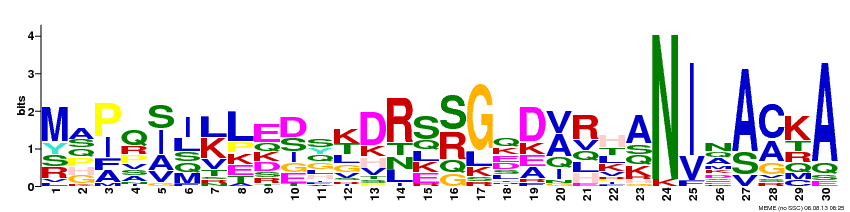


MA[PI]Q[ISA][IL][KL]LE[DS]S[KT]DRS[SR]GQD[AV]RHAN[IV]N[AS][CA]KA

## Motif 17

E-value 1.3e-088

Width 42

Sites 11


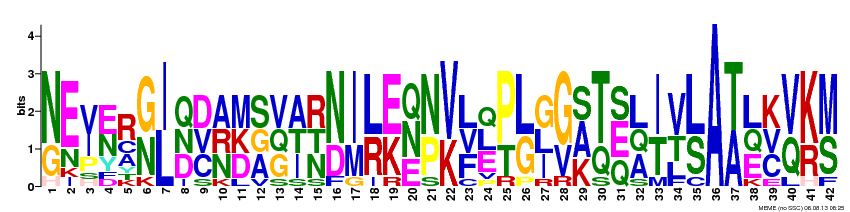


[NG]E[VI][EN]R[GN][IL][QDN][DCV][ANR][MDK][SAG][VGQ][AIT][RNT][ND][IM][LR][EK][NQE][NP][VK][LFV][ELQ][PT][LG][GIL][GV][ASK][TQ][ESQ][LAQ][IT][VT][LS]A[TA][LEQ][KCV][VQ][KR][MS]

## Motif 18

E-value 2.7e-021

Width 30

Sites 5


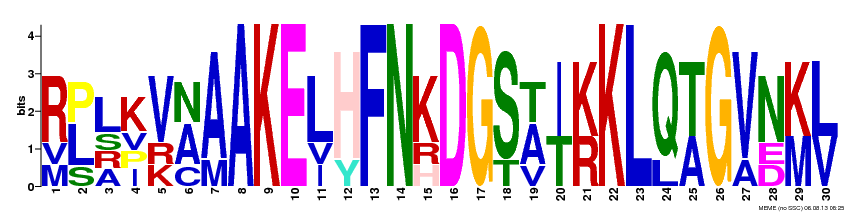


[RMV][LPS][LARS][KIPV][VKR][ANC][AM]AKE[LIV][HY]FN[KHR]DG[ST][ATV][IT][KR]KL[QL][TA]G[VA][NDE][KM][LV]

## Motif 19

E-value 2.0e+004

Width 30

Sites 2


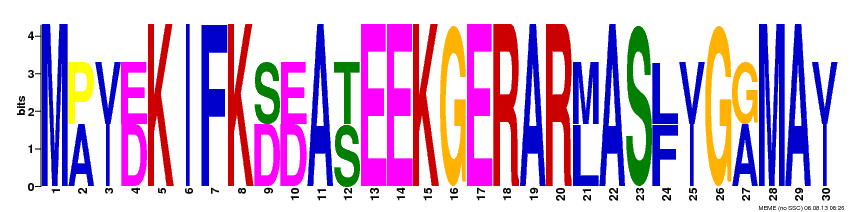


M[AP][IV][DE]KIFK[DS][DE]A[ST]EEKGERAR[LM]AS[FL][IV]G[AG]MA[IV]

## Motif 20

E-value 7.0e+004

Width 30

Sites 2


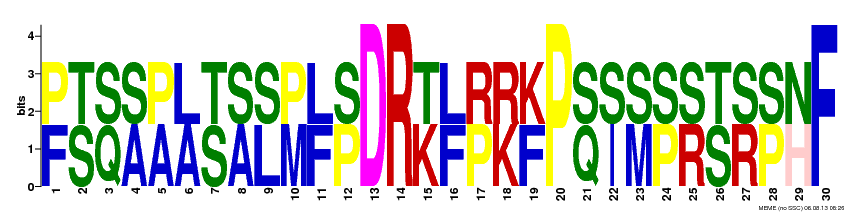


[FP][ST][QS][AS][AP][AL][ST][AS][LS][MP][FL][PS]DR[KT][FL][PR][KR][FK]P[QS][IS][MS][PS][RS][ST][RS][PS][HN]F
