# Supplementary material for: Hsf and Hsp gene families in Populus: genome-wide identification, organization and correlated expression during development and in stress responses
Source: BMC Genomics. 2015 Mar 14;16(1):181. doi: 10.1186/s12864-015-1398-3 (PMC4373061; doi:10.1186/s12864-015-1398-3)
Supplement: Additional file 7: Table S7. — Sequence logos for the conserved motifs of Hsp70 proteins in Arabidopsis and Populus. [file 12864_2015_1398_MOESM7_ESM.docx]

**Table S7. Sequence logos for the conserved motifs of Hsp70 proteins in *Arabidopsis* and *Populus*.**

**Hsp70 Motif**

**Motif 1**

E-value 9.9e-1705

Width 70

Sites 34


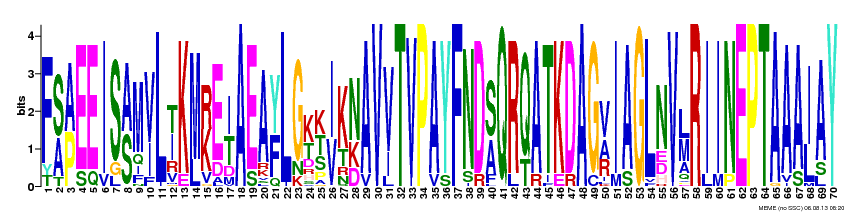


F[SA][AP]EEIS[AS]M[VI]L[TI]K[ML][RK]E[IT]AEA[YF]LG[KT][KTS][IV]K[NK]AV[VI]TVPAYFND[SA]QR[QT]ATKDAG[VAR]IAGLNV[LM]RIINEPTAAA[IL]AY

## Motif 2

E-value 1.1e-1708

Width 70

Sites 34


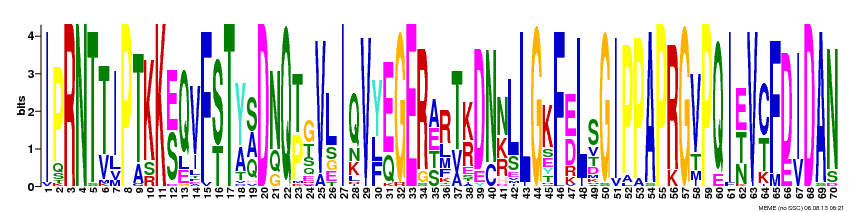


IPRNT[TV]IPTKK[ES]QVF[ST]T[YA][SAQ]D[NQ]Q[TP][GT]VLIQV[YFL][EQ]GER[AEST]R[TV][KR]DN[NKR]LLGKF[ED]LSGIPPAPRGVPQI[ETN]V[CT]F[DE][IV]DAN

## Motif 3

E-value 3.6e-1528

Width 70

Sites 31


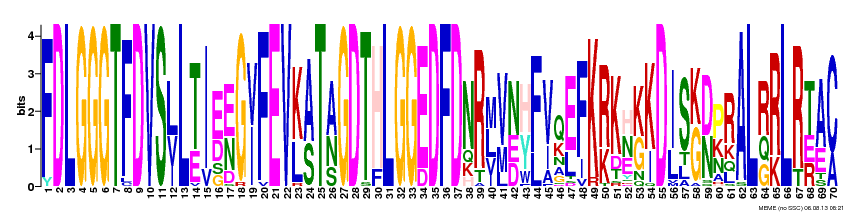


FDLGGGTFDVS[LV]L[TE]I[ED][EN]G[VI]FEV[KL][AS]T[AN]GDTHLGGEDFDNR[ML][VM][NED][HY][FL][VI]Q[EL]FK[RK]K[HNE][KG][KI]D[IL]S[GK][DN][PK]RAL[RQG][RK]LR[TER][AE][CA]

## Motif 4

E-value 2.7e-1502

Width 69

Sites 50


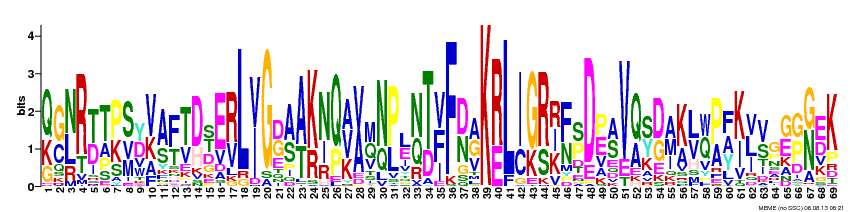


[QK][GC][NL]R[TD]T[PK][SVM][YD][VK][AS][FT][TV][DH][ST]E[RV]L[IV]G[DG][AS][AT][KR][NI][QP][AVK][VA][MQ][NQ][PL][LE][NQ][TD][VFI]F[DN][AGV]K[RE]L[IC][GK][RS][RIK][FN][SP]D[PE][AS]V[QA][SY][DG][AMI][KA][LV][QW][PA][FAY][KI][VLI][VS]G[GE][GP][GN][ED]K

## Motif 5

E-value 3.1e-1087

Width 70

Sites 23


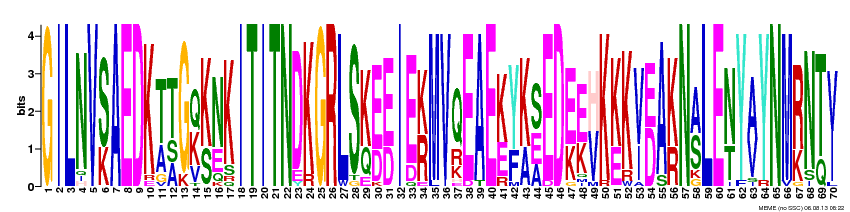


GILNV[SK]AEDK[TA][TAS]G[QK][KS][NE]KITITNDKGRLS[KQ][ED][ED]IE[KR]MVQEAE[KE][YF][KA][SEA]ED[EK][EK][HV]K[KE]K[VI][ED]A[KR]N[AS]LE[NT]Y[AV]YNM[RK]N[TQ][VI]

## Motif 6

E-value 2.2e-797

Width 70

Sites 22


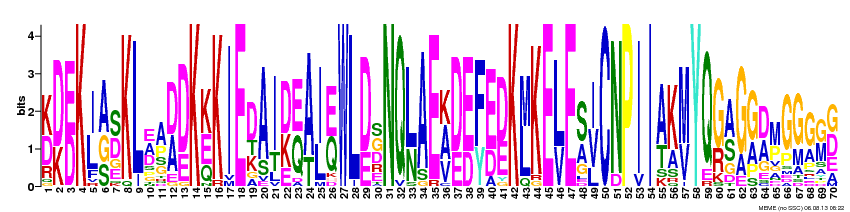


[KD][DK][ED]K[IL][AGS][SD]KL[AE]A[DA][DE]K[KE]KIEDAI[DK][EQ][AT][IL][EQ]WL[DE][GS]NQ[LN]AE[AKV][DE][ED][FY][ED][DE]K[LM]KE[LV]E[SA][IV]CNPII[AT]K[MV]YQ[GR][AS]G[GA][DA][MP]GGG[GM][GD]

## Motif 7

E-value 1.1e-734

Width 42

Sites 33


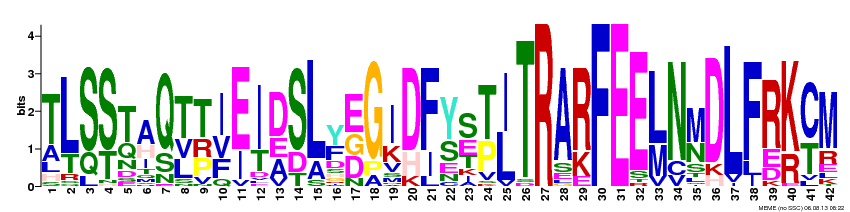


TL[SQ][ST][TQ][AH]Q[TLV][TPR][IVF][EI][IT][DAE]SL[YF][EDG]G[IK][DH][FI][YS][SE][TP][IL]TRA[RK]FEE[LM]N[MN]DLFR[KR][CT]M

## Motif 8

E-value 2.8e-614

Width 30

Sites 34


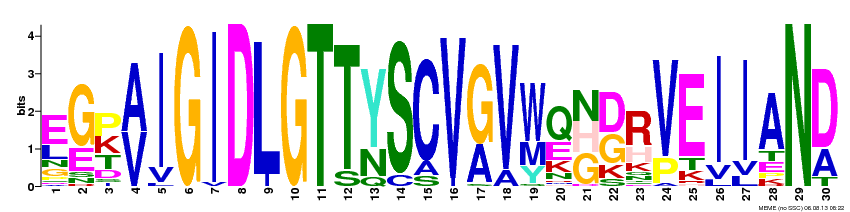


[EL][GE][PKT][AV]IGIDLGTT[YN]SCV[GA]V[WM][QE][GHN][DG][RH][VP]EIIAN[DA]

## Motif 9

E-value 4.8e-550

Width 59

Sites 21


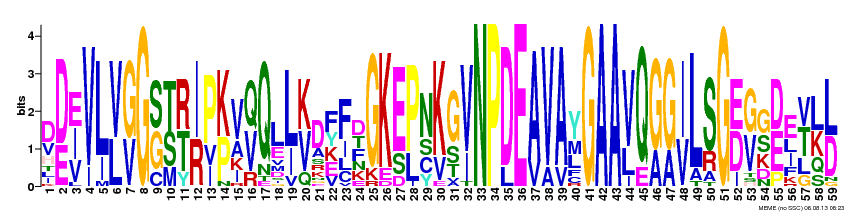


D[DE][EIV]VL[VL][GV]G[SG][TS][RT][IR]P[KP]V[QV]QL[LI][KV]DFFDGKE[PL]NKG[VI]NPDEAVAYGAA[VL]Q[GA][GA][IV]LSG[ED][GV][GS][DE]E[TV][LK][LD]

## Motif 10

E-value 1.4e-092

Width 70

Sites 5


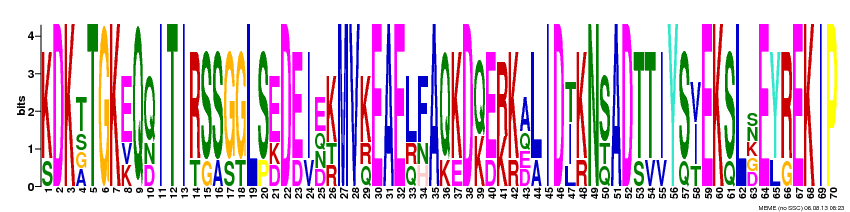


[KS]DK[TAGS]TGK[EKV]Q[QDN]ITI[RT][SG][SA][GS][GT]L[SP][EDK]D[ED][IV][EDNQ][KRT]MV[KQR]EAE[LQR][FHN]A[QK][KE]D[QK][ED][RK][KR][ADEQ][LA]ID[ITL][KR]N[SQT]AD[TS][TV][IV]Y[SQ][IVT]EK[SQ]L[DGKNS]E[YL][RG]EKIP

## Motif 11

E-value 2.3e-065

Width 52

Sites 10


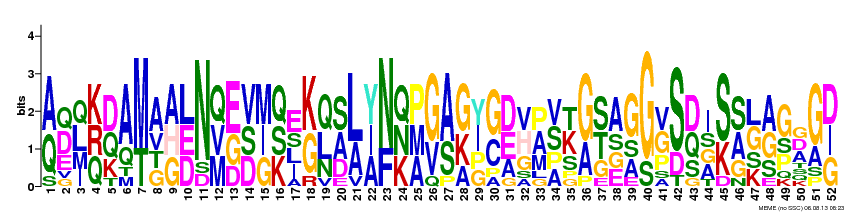


[AQ][DQE][LQIM][KQR][DQK][AQ][MT][AGTV][AGH][LDE]N[QMV][EGD][VDS][MGI][QKS][EILS][KG][QLN][SAD][LA][YAI][NF][QKN][PAM][GV][AS][GKA][IYGP][GC][DEA][HVG][APM][SVAP][KTS][GA][STG][AEGS][GAS][GS][GVP][SD][DQS][IAGS][SK][SAG][GLKS][AGS][GPS][ADG][GA][DGI]

## Motif 12

E-value 3.5e-034

Width 42

Sites 3


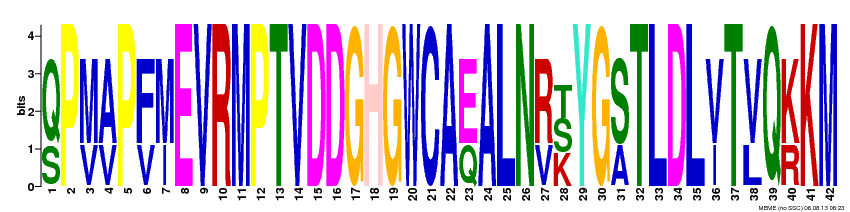


[QS]P[MV][AV]P[FV][MI]EVRMPTVDDGHGWCA[EQ]ALN[RV][KST]YG[SA]TLDL[VI]T[VL]Q[KR]KM

## Motif 13

E-value 5.2e-026

Width 30

Sites 5


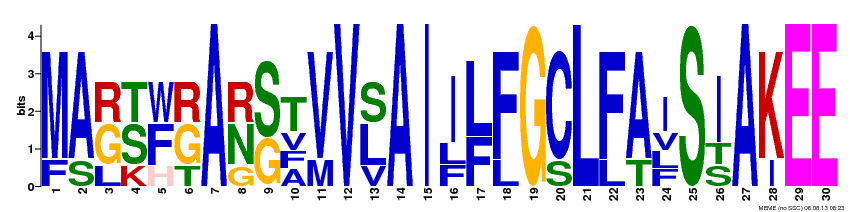


[MF][AS][GRL][STK][FWH][GRT]A[NRG][SG][TAFV][VM]V[LSV]AI[IFL][LF][FL]G[CS]L[FL][AT][IFLV]S[IST]A[KI]EE

## Motif 14

E-value 5.0e-022

Width 33

Sites 4


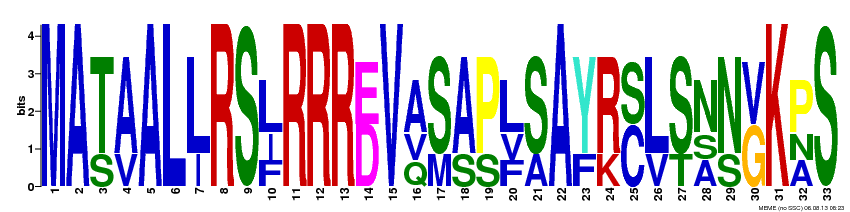


MA[TS][AV]AL[LI]RS[LFI]RRR[DE]V[AQV][SM][AS][PS][LFV][SA]A[YF][RK][CS][LV][ST][NAS][NS][GV]K[PAN]S

## Motif 15

E-value 1.8e-015

Width 36

Sites 4


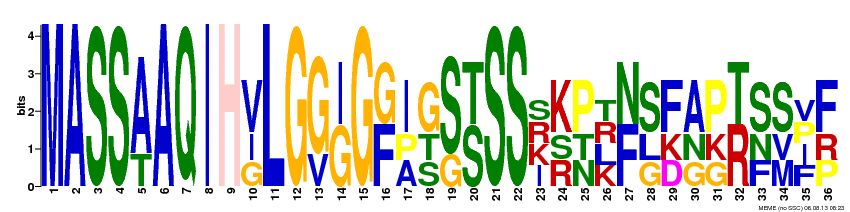


MASS[AT]AQIH[VGI]LG[GV][GI]G[FG][IAP][GST][SG][ST]SS[IKRS][KRS][PNT][KLRT][FN][SGL][FDK][AGN][PGK][RT][SFN][SMV][FIPV][FPR]

## Motif 16

E-value 8.4e-008

Width 30

Sites 4


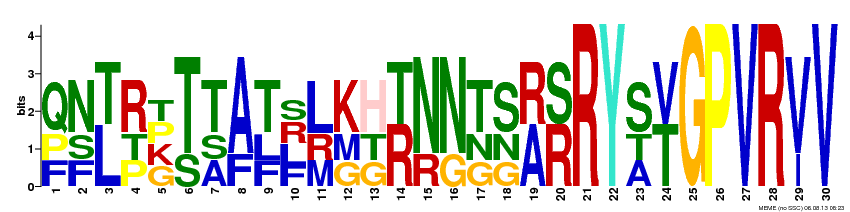


[QFP][NFS][LT][RPT][GKPT][TS][TAS][AF][TFL][FLRS][LMR][KGM][HGT][RT][NR][NG][TGN][SGN][AR][RS]RY[SAT][TV]GPVR[VI]V

## Motif 17

E-value 1.0e-007

Width 42

Sites 2


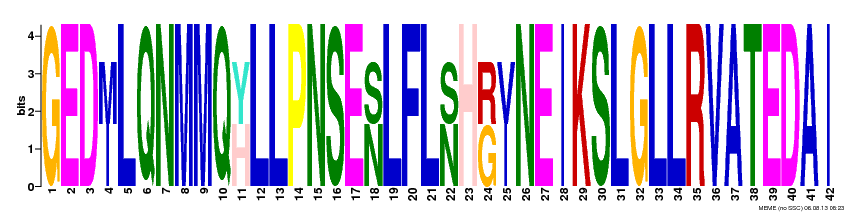


GED[IM]LQNMMQ[HY]LLPNSE[NS]LFL[NS]H[GR][IV]NEIKSLGLLRVATEDAI

## Motif 18

E-value 2.1e-010

Width 30

Sites 3


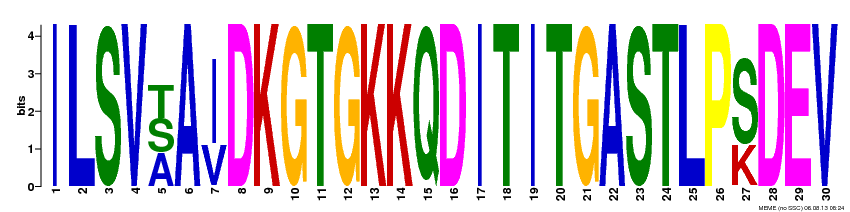


ILSV[AST]A[IV]DKGTGKKQDITITGASTLP[SK]DEV

## Motif 19

E-value 4.1e+006

Width 35

Sites 2


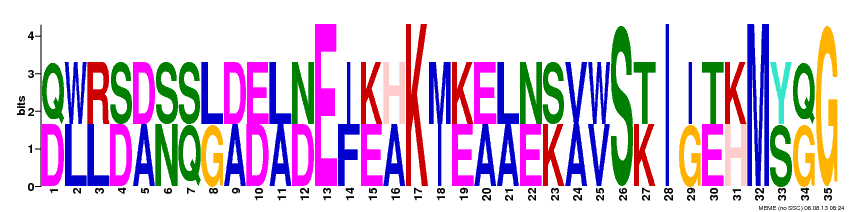


[DQ][LW][LR][DS][AD][NS][QS][GL][AD][DE][AL][DN]E[FI][EK][AH]K[IM][EK][AE][AL][EN][KS][AV][VW]S[KT]I[GI][ET][HK]M[SY][GQ]G

## Motif 20

E-value 5.7e+006

Width 30

Sites 2


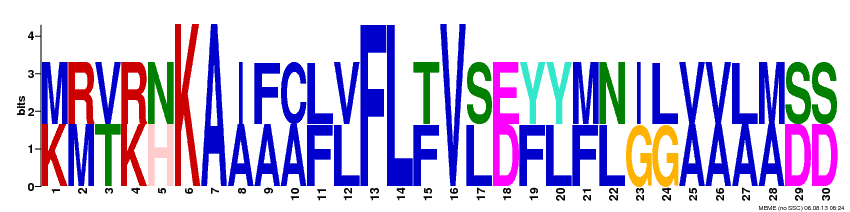


[KM][MR][TV][KR][HN]KA[AI][AF][AC][FL][LV]FL[FT]V[LS][DE][FY][LY][FM][LN][GI][GL][AV][AV][AL][AM][DS][DS]
