# Supplementary material for: Hsf and Hsp gene families in Populus: genome-wide identification, organization and correlated expression during development and in stress responses
Source: BMC Genomics. 2015 Mar 14;16(1):181. doi: 10.1186/s12864-015-1398-3 (PMC4373061; doi:10.1186/s12864-015-1398-3)
Supplement: Additional file 8: Table S8. — Sequence logos for the conserved motifs of Hsp100 proteins in Arabidopsis and Populus. [file 12864_2015_1398_MOESM8_ESM.docx]

**Table S8. Sequence logos for the conserved motifs of Hsp100 proteins in *Arabidopsis* and *Populus*.**

**Hsp100 Motif**

**Motif 1**

E-value 1.3e-349

Width 42

Sites 18


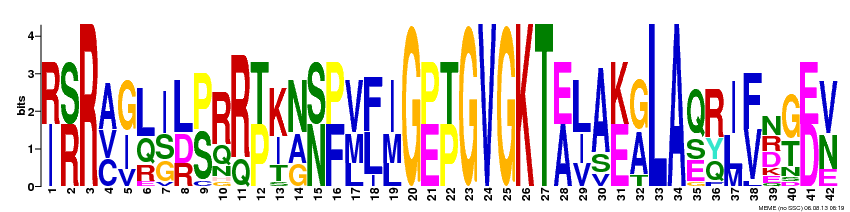


[IR][RS]R[ACV][GI][LQ][ISG][LDR][PS][RQ][RQ][PT][KI][NAG][NS][FP][VML][FL][IML]G[EP][PT]GVGKT[AE][LIV]A[EK][GA]LA[QES][RQY][IL][FV][NDR][GT][DE][VN]

## Motif 2

E-value 1.0e-420

Width 59

Sites 17


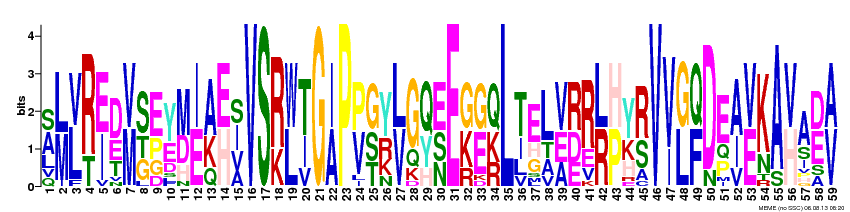


[AS][LM][LV][RT][EI][DE][VM][SGT][EP][YE][MD][IE][AK][EH][ISV]VS[RK][WL][TI]G[IA]P[PV][GST][YR][LV]G[QY][ESN]E[GK][GE][QKR]L[TI]EL[VEA][RDE]R[LR][HP][YK][RS]V[VI][GL][QF]DE[AI][VE]KA[VH]A[DE][AV]

## Motif 3

E-value 1.3e-377

Width 70

Sites 9


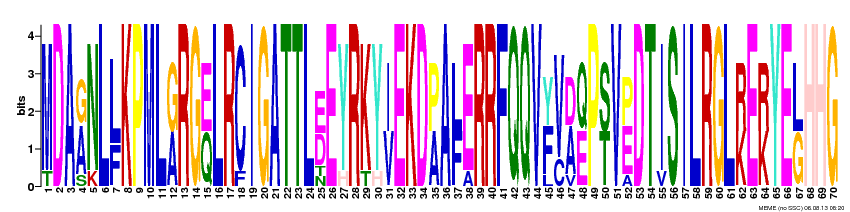


MDA[AG]NL[LF]KPML[GA]RG[EQ]LRCIGATTL[ED]EYRKY[IV]EKD[PA]A[LF]ERRFQQV[FY][VC][AD][QE]P[ST]V[EP]DTISILRGL[RK]E[RK]YE[LG]HHG

## Motif 4

E-value 1.8e-261

Width 70

Sites 9


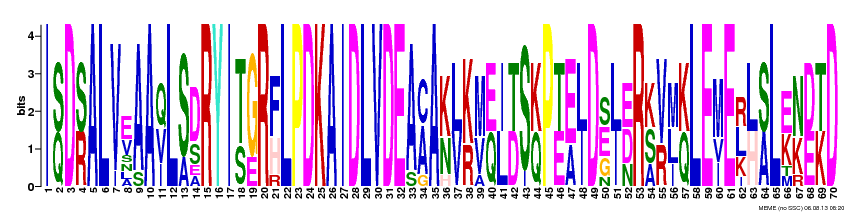


I[SQ]D[SR]ALV[EV]AA[QVI]L[SA][DS]RYI[TS][GE]R[FH]LPDKAIDLVDEA[AC]A[KN][LV][KR][MV][EQ][IL][TD]S[KQ]P[TE][EA][LI]D[ESG][LI][EDN]R[KSA][VR][MLI][KQ]LE[MIV]E[LRK][LH][SA]L[EK][NK][DE][TK]D

## Motif 5

E-value 1.5e-240

Width 59

Sites 9


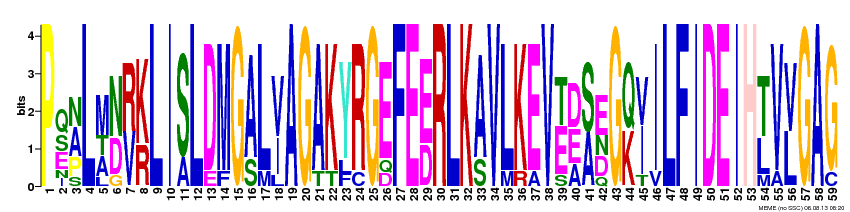


P[QES][ANP]L[MAT][ND][RV][KR]LI[SA]LDMG[AS]L[VI]AGAKYRGEFE[ED]RLK[AS]VLKEV[ET][DEA][SA][EDN]G[QK][IV]ILFIDEIH[TL]V[VL]GAG

## Motif 6

E-value 8.0e-205

Width 70

Sites 8


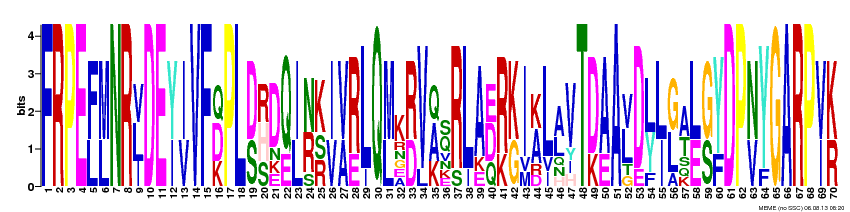


FRPE[FL][ML]NR[LVI]DE[YI][IV]VF[DQK]PL[DS][HRS]D[QE][IL][NR][KRS][IV][VA][RE]LQ[ML]K[RD]V[AQK][QS]RLA[DEQ][RK][KG]I[AK]L[AL]VT[DK][AE]A[LV]D[LY][LI][GL][AT][LE][GS][YF]DP[NV]YGARP[VI][KR]

## Motif 7

E-value 9.3e-200

Width 70

Sites 9


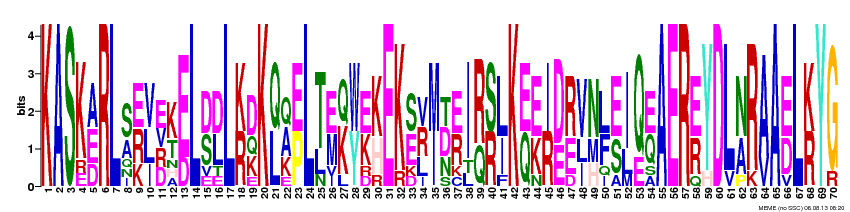


KASK[AED]RL[SA][ERK][VLI][EDRV][KT][ED]L[DS][DL]L[KR][DKQ]K[QL][AQK][EP]L[TL][EM][KQ][WY][EK][KH]EK[SE][RVL][MI][DTN][ER]I[RQ][SR][LI]K[EQ][EK][IR][DE][RE][VIL][NHM][LF][ES][IL][QE][EQ]AER[ER]YDL[NA]R[AV]A[ED]L[KR]YG

## Motif 8

E-value 3.2e-159

Width 42

Sites 8


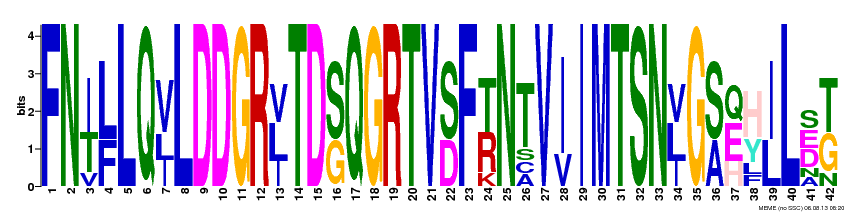


FN[IT][LF]LQ[VIL]LDDGR[LVI]TD[SG]QGRTV[SD]F[TR]NTV[IV]IMTSN[LVI]G[SA][EQH][HY][IL]L[DES][TG]

## Motif 9

E-value 4.1e-145

Width 70

Sites 8


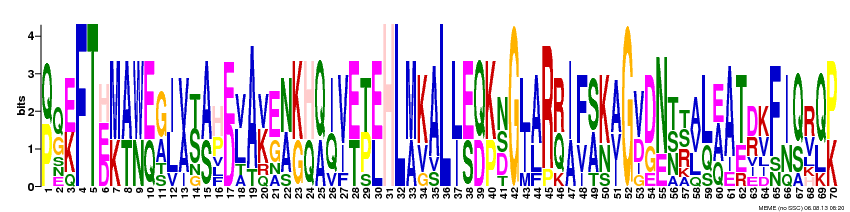


[PQ][QG][EK]FT[EHD][MK][AT][WN][EQ][GA][IL][VA][ST][AS][HP][DE][LV]A[KV][EG][NA][KG][HQ][QA][IQ][VI][ET][TP][EL]HL[MA][KV]AL[LI][ES][QD][KP][NDS]G[LI][AL]R[RQ][IA][FV][SA][KN][AIV]G[VD][DG][NE][TNS][RST][ALV][LQ][AEQ]A[TE][DR][KV]F[IN][QS][RV][QL][PK]

## Motif 10

E-value 6.6e-112

Width 59

Sites 8


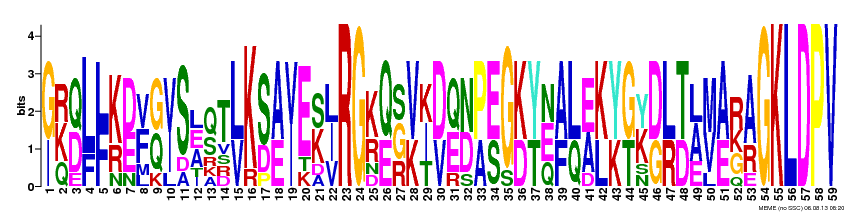


[GI][KRQ][QD][LF][FL][KR][DE][FV][GQ][VI]S[AEL][QS]T[LV]K[SD][AE][IV]E[KS][ILV]RG[KR][QE][GSR][VK][IKT][DV][QE][ND][PA][ES]G[KD][YT][ENQ][AF][LQ][EA][KL][YK][GT][KY][DG][LR][TD][ALE][MV][AE][RGK][AR]GKLDPV

## Motif 11

E-value 2.6e-050

Width 30

Sites 8


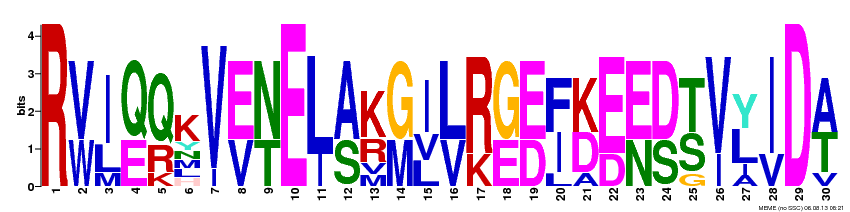


R[VW][IL][QE][QR]KV[EV][NT]E[LI][AS][KR][GM][ILV][LV][RK][GE][ED][FI][KD][ED][EN][DS][TS][VI][LY][IV]D[AT]

## Motif 12

E-value 6.7e-053

Width 42

Sites 9


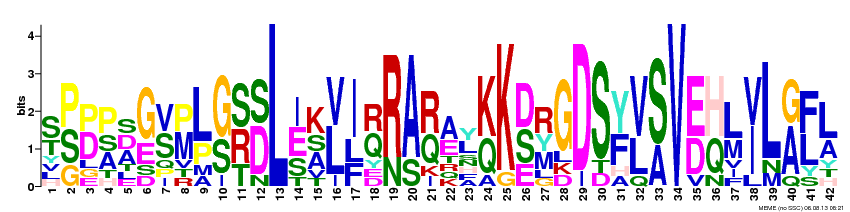


[ST][PSG][DP][PAS][ADS][GE][SV][MP][LP][GS][SRT][DS]L[EIS][KAS][LV][IFL][QR][RN][AS][RQ][AE][LY][KQ]K[DSE][RMY]GDS[YF][VL][SA]V[ED][HQ][LM][IV]L[AG][FL][LA]

## Motif 13

E-value 2.1e-016

Width 59

Sites 3


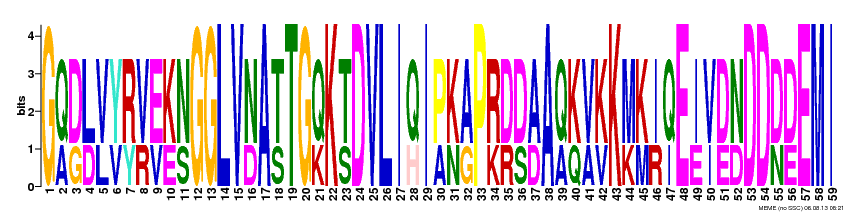


G[QA][DG][LD][VL][YV][RY][VR][EV][KE][NS]GGLV[ND]A[TS]TG[QK]K[TS]DVLI[QH]I[PA][KN][AG]P[RK][DR][DS][AD]A[QA][KQ][VA][KV]K[MK][KM][IR][QI]E[IE][VI][DE][ND]DD[DN][DE]EMI

## Motif 14

E-value 1.8e-007

Width 30

Sites 3


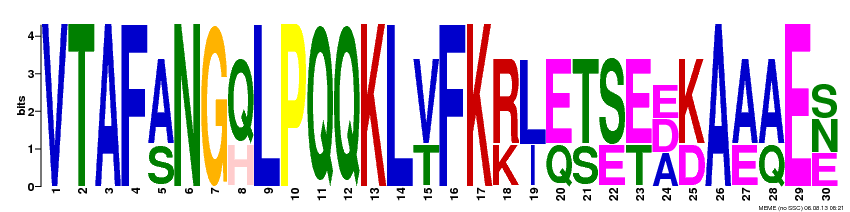


VTAF[AS]NG[QH]LPQQKL[VT]FK[RK][LI][EQ][TS][SE][ET][ADE][KD]A[AE][AQ]E[ENS]

## Motif 15

E-value 2.7e-006

Width 69

Sites 3


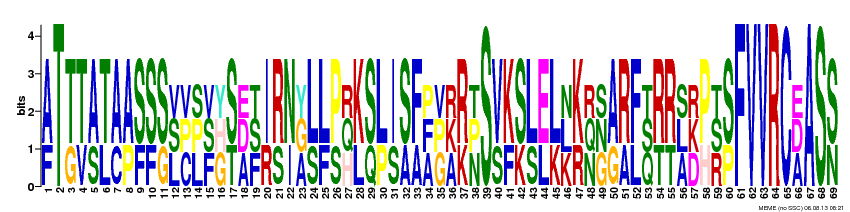


[AF]T[TG][TV][AS][TL][AC][AP][SF][SF][SG][LSV][CPV][LPS][FSV][GHY][ST][ADE][FST][IR][RS][NI][AGY][LS][LF][PS][HQR][KL][SQ][LP][IS][SA][FA][AFP][GPV][AKR][RK][NPT]S[VS][KF][SK][LS][EL][LK][KLN][KR][NQR][GNS][AG][RA][FL][QST][RT][RT][ALS][DKR][PH][RST][SP]FVVRC[ADE]AS[SN]

## Motif 16

E-value 4.1e+001

Width 40

Sites 2


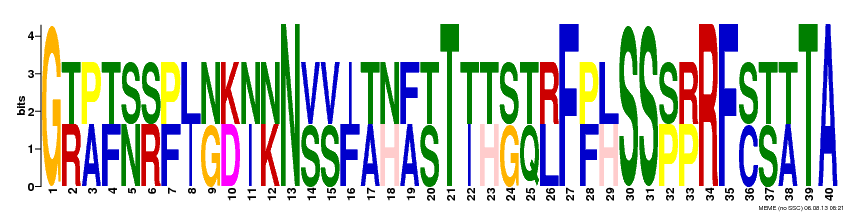


G[RT][AP][FT][NS][RS][FP][IL][GN][DK][IN][KN]N[SV][SV][FI][AT][HN][AF][ST]T[IT][HT][GS][QT][LR]F[FP][HL]SS[PS][PR]RF[CS][ST][AT]TA

## Motif 17

E-value 5.1e+004

Width 30

Sites 2


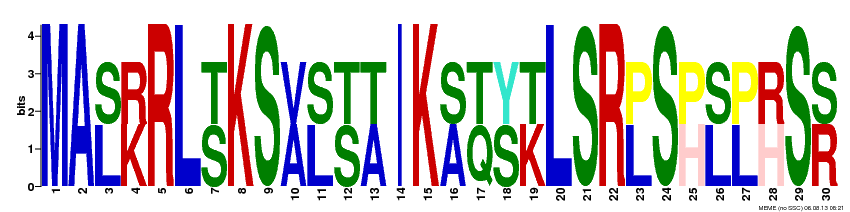


MA[LS][KR]RL[ST]KS[AV][LS][ST][AT]IK[AS][QT][SY][KT]LSR[LP]S[HP][LS][LP][HR]S[RS]

## Motif 18

E-value 2.5e+006

Width 30

Sites 2


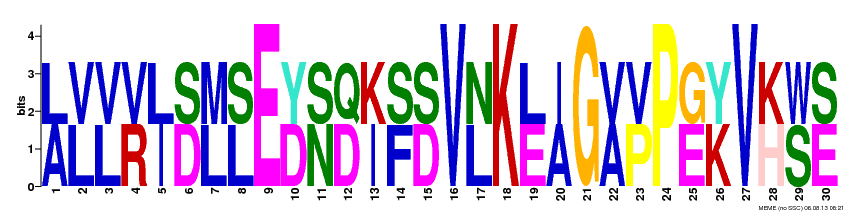


[AL][LV][LV][RV][IL][DS][LM][LS]E[DY][NS][DQ][IK][FS][DS]V[LN]K[EL][AI]G[AV][PV]P[EG][KY]V[HK][SW][ES]

## Motif 19

E-value 2.8e+007

Width 30

Sites 2


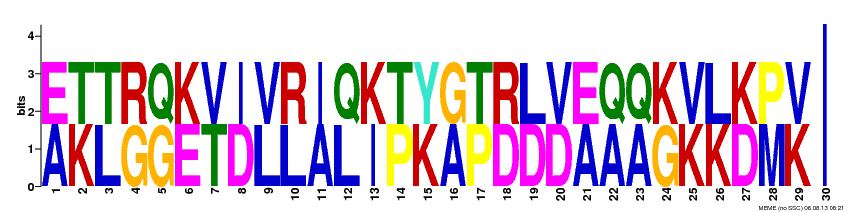


[AE][KT][LT][GR][GQ][EK][TV][DI][LV][LR][AI][LQ][IK][PT][KY][AG][PT][DR][DL][DV][AE][AQ][AQ][GK][KV][KL][DK][MP][KV]I
