# Supplementary figures and images for: Hsf and Hsp gene families in Populus: genome-wide identification, organization and correlated expression during development and in stress responses
Source: BMC Genomics. 2015 Mar 14;16(1):181. doi: 10.1186/s12864-015-1398-3 (PMC4373061; doi:10.1186/s12864-015-1398-3)

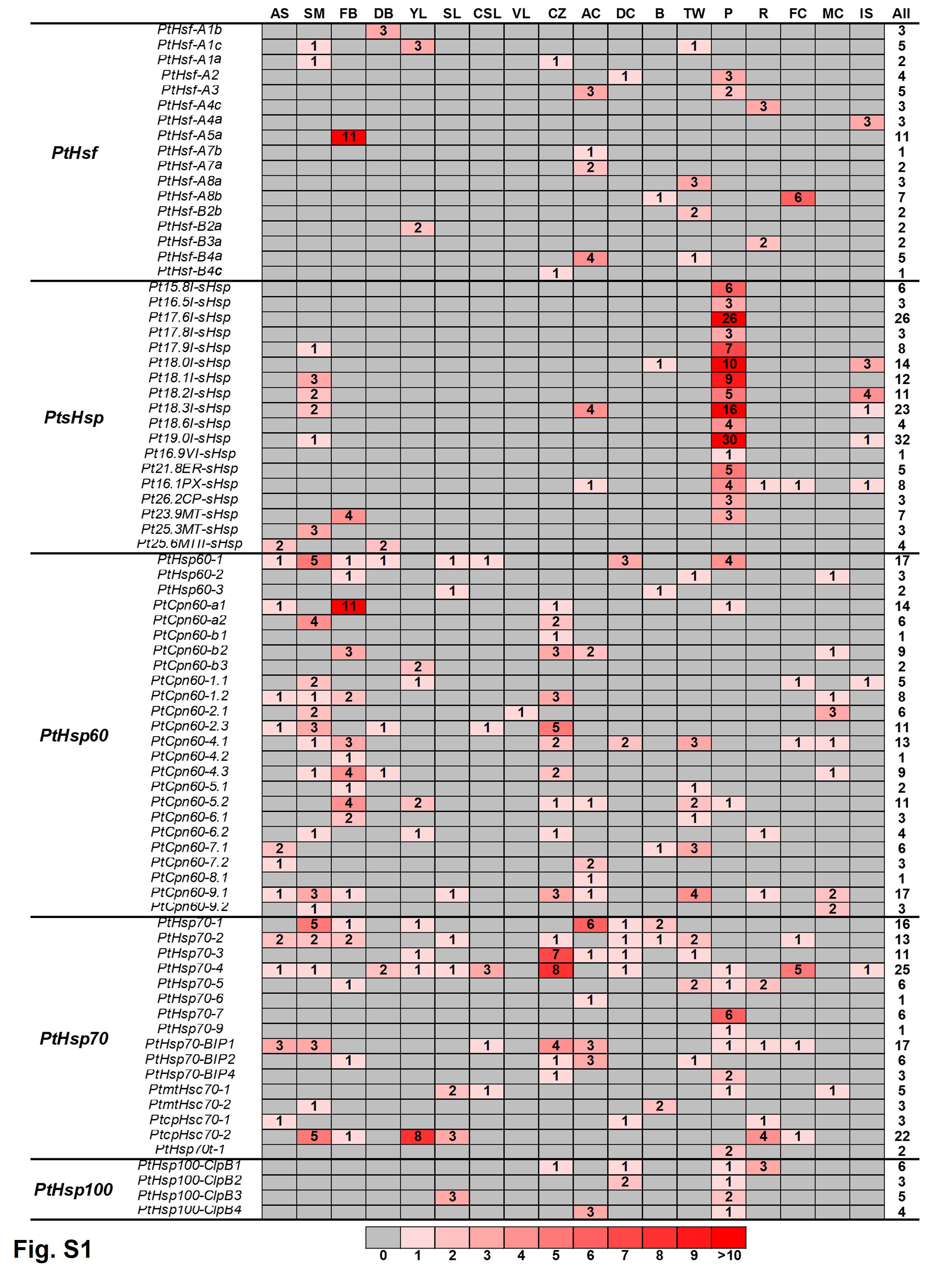

Supplement: Additional file 9: Figure S1. — In silico EST analysis of Populus Hsfs and Hsps. EST frequency for each gene was calculated by evaluating its EST representation among 17 cDNA libraries available at PopGeneIE (http://popgenie.org/) [43]. Color bar at bottom represents the frequencies of EST counts. P: petioles, IS: imbibed seeds, B: bark, AC: active cambium, FB: flower buds, TW: tension wood, MC: male catkins, DC: dormant cambium, AS: apical shoot, DB: dormant buds, FC: female catkins, CZ: cambial zone, CSL: cold stressed leaves, SL: senescing leaves, YL: young leaves, R: roots, SM: shoot meristem. [file 12864_2015_1398_MOESM9_ESM.jpeg]
